# Quality appraisal of clinical practice guidelines for motor neuron diseases or related disorders using the AGREE II instrument

**DOI:** 10.3389/fneur.2023.1180218

**Published:** 2023-07-17

**Authors:** Jia-Yin Ou, Jun-Jun Liu, Jing Xu, Jia-Yu Li, Yang Liu, You-Zhang Liu, Li-Ming Lu, Hua-Feng Pan, Lin Wang

**Affiliations:** ^1^South China Research Center for Acupuncture and Moxibustion, Medical College of Acu-Moxi and Rehabilitation, Guangzhou University of Chinese Medicine, Guangzhou, China; ^2^Science and Technology Innovation Center, Guangzhou University of Chinese Medicine, Guangzhou, China; ^3^The First Affiliated Hospital of Guangzhou University of Chinese Medicine, Guangzhou, China; ^4^The Second School of Clinical Medicine, Guangzhou University of Chinese Medicine, Guangzhou, China

**Keywords:** AGREE II, clinical practice guidelines, critical appraisal, motor neuron disease, quality assessment, spinal muscular atrophy

## Abstract

**Objectives:**

This study aimed to systematically assess the quality of CPGs for motor neuron diseases (MNDs) or related disorders and identify the gaps that limit evidence-based practice.

**Methods:**

Four scientific databases and six guideline repositories were searched for eligible CPGs. Three researchers assessed the eligible CPGs using the Appraisal of Guidelines Research and Evaluation II instrument. The distribution of the level of evidence and strength of recommendation of these CPGs were determined. The univariate regression analysis was used to explore the characteristic factors affecting the quality of CPGs.

**Results:**

Fifteen CPGs met the eligibility criteria: 10 were for MND and 5 were for spinal muscular atrophy. The mean overall rating score was 44.5%, and only 3 of 15 CPGs were of high quality. The domains that achieved low mean scores were applicability (24.4%), rigor of development (39.9%), and stakeholder involvement (40.3%). Most recommendations were based on low-quality evidence and had a weak strength. The CPGs that were updated, meant for adults, and evidence based, and used a CPG quality tool and a grading system were associated with higher scores in certain specific domains and overall rating.

**Conclusion:**

The overall quality of CPGs for MNDs or related disorders was poor and recommendations were largely based on low-quality evidence. Many areas still need improvement to develop high-quality CPGs, and the use of CPG quality tools should be emphasized. A great deal of research on MNDs or related disorders is still needed to fill the large evidence gap.

## 1. Introduction

Motor neuron diseases (MNDs) or related disorders, such as MND, spinal muscular atrophy (SMA), and post-polio progressive muscular atrophy (PPMA), are a group of disorders characterized by progressive weakness secondary to degeneration of the motor neurons (1). MND is caused by the loss of motor neurons leading to muscle atrophy, paralysis, and ultimately death within 3–5 years after the onset of the disease (2). Although the treatment options for MND are limited and patient care primarily focuses on controlling symptoms and optimizing functioning and quality of life (3), better multidisciplinary care and a better understanding of interventions may allow patients with MND to live longer life (4). The data from the Global Burden of Disease study show that the burden of MND is increasing (5).

SMA is an autosomal recessive disease caused by biallelic mutations in the survival motor neuron 1 gene, causing progressive muscle weakness and atrophy (6). It imposes a significant burden on patients, caregivers, and the healthcare system (7), but the financial burden could be significantly reduced by expanding newborn screening for SMA in combination with early treatment interventions for newborns with SMA (8). For example, newborn screening for patients with SMA is associated with earlier diagnosis and intervention, and motor milestones are often achieved at an earlier age, compared with clinically diagnosed patients (8). PPMA, also known as post-polio syndrome (PPS), is a chronic progressive disorder that may appear decades after the initial acute polio infection (9), affecting 20–40% of polio survivors and manifesting as neuromuscular complications (10). Although the incidence of paralytic poliomyelitis has declined significantly (11), PPS will remain a major health problem for many years (12). At present, the diagnosis and management of MNDs or related disorders remain a challenge for clinicians and vary widely in practice.

One of the foundations of improving healthcare work are clinical practice guidelines (CPGs) (13). CPGs are defined as “statements that include recommendations intended to optimize patient care that are informed by a systematic review of evidence and an assessment of benefits and harms of alternative care options” (14). CPGs help improve the quality of health care, such as informing clinicians about treatment decisions for patients and determining the appropriate standard of care, thus identifying gaps between evidence and practice (15).

The usefulness of CPGs depends primarily on quality, rigorous methodology, and transparent development processes (16). Better CPG quality appears to be associated with more positive treatment outcomes (17). Some common problems with CPGs include the sheer volume available, large amount of documentation that is difficult to assimilate or use, lack of clear supporting evidence, exclusion of key stakeholders, insufficient editorial independence, and poor applicability (18–20). Besides, determining the level of evidence on which the recommendations are based is important (21, 22). However, the systemic appraisal of CPGs for MNDs or related disorders remians unreported, and the distribution of the level of evidence on which these recommendations are based has not been described.

Various tools have been developed to assess the quality of CPGs (23). The Appraisal of Guidelines Research and Evaluation (AGREE) Instrument was first published in 2003 and updated in 2009 (AGREE II). It is designed to assess the methodological quality of CPGs and also provide methodological strategies for the development of new CPGs (24). The AGREE II instrument is currently the preferred tool for the quality assessment of CPGs in the world and can be used to assess CPGs of multiple diseases (15, 24–27).

Therefore, the present study aimed to assess the quality of CPGs for MNDs or related disorders using the AGREE II instrument, identify the distribution of the level of evidence and strength of recommendations of these CPGs, and also identify the potential influencing factors for the quality of CPG. The findings of this study would help identify the gaps that hinder evidence-based practice and highlight potential opportunities for improvement.

## 2. Materials and methods

### 2.1. Eligibility criteria

CPGs were included if they: (1) focused on the diagnosis and management of MNDs or related disorders, including MND, SMA, and PPMA (1); (2) were published between January 1, 2006, and September 4, 2022; and (3) were written in English. Consistent with previous studies (25, 26), both evidence-based and consensus-based CPGs were included. If the CPG was updated, the latest version was included.

CPGs were excluded if (1) full texts were unavailable; (2) they were editorials, comments, reviews, letters, and correspondence studies; (3) they were interpretation, translation, and adaptation of CPGs; and (4) they were duplicate publications.

### 2.2. Literature search

Scientific databases, including PubMed, Embase, Cumulative Index to Nursing and Allied Health Literature, and Physiotherapy Evidence Database PEDro, were systematically searched under the direction of a reference librarian. Besides, the following six online guideline repositories were also searched: National Institute for Health and Clinical Excellence (NICE), Scottish Intercollegiate Guidelines Network, Guidelines International Networks, Agency for Healthcare Research and Quality, National Health and Medical Research Council, and World Health Organization. Keywords and Medical Subject Headings related to CPGs and MNDs or related disorders were combined in the database search. The specific search strategy is displayed in Supplementary File 1. The search range was from January 1, 2006, to September 4, 2022.

### 2.3. Study selection and data extraction

Two researchers (Jia-yin Ou and Jun-Jun Liu) independently performed the study selection and data extraction. Any disagreements between the two were resolved through discussion or consultation with a third researcher (Jing Xu). For study selection, the search results were first exported into the EndNote X7 literature management software (Thomson Reuters Corporation, CA, United States), excluding duplicates. Two researchers reviewed the titles and abstracts of the studies for screening to exclude the explicitly irrelevant studies, and then read the full texts of the remaining studies to determine whether they were finally included. For each CPG included in the end, the accompanying technical and supporting documents were thoroughly searched to better inform our evaluation.

The data were extracted into a specially designed spreadsheet. The extracted variables included the year of publication, disease, development organization, first author (if applicable), country/region of origin, version, age range of target population (adult/children/all ages), development method, search dates covered, CPG quality tool used, CPG methodologist included, title, funding sources, and accompanying documents. The country/region of origin was described as “Europe” if the CPG was jointly developed by multiple European countries, and “international” if it was jointly developed by multiple countries from different continents. The development method was described as “evidence based” if the CPG performed a systematic search and evaluation of evidence and made recommendations based on the evaluation results during the CPG development process; otherwise, it was described as “consensus based.”

### 2.4. Quality assessment

Three researchers (Jia-Yin Ou, Jun-Jun Liu, and Jing Xu) assessed the quality of each CPG independently using the AGREE II tool under the guidance of a methodological expert (Liming Lu). The AGREE II tool included 23 key items organized in 6 domains followed by 2 global rating items (“Overall Assessment”). The six domains included scope and purpose, stakeholder involvement, rigor of development, clarity of presentation, applicability, and editorial independence. Each item was rated on a 7-point scale, with 1 indicating strongly disagree and 7 indicating strongly agree. Strongly disagree meant no information was relevant to an item, and strongly agree meant the quality of reporting was exceptional and the full criteria and considerations were met for an item. A score between 2 and 6 was assigned when the reporting did not meet the full criteria or considerations for an item. According to the calculation formula, the scores in each domain were calculated and the calculation formula was as follows: each domain score = (obtained score – minimum possible score)/ (maximum possible score – minimum possible score) × 100%. Consistent with a previous study (28), the score of a domain or overall rating ≤ 40% was considered as a low rating, >40 and ≤ 70% as a moderate rating, and > 70% as a high rating.

In the overall assessment, the first global rating item (“overall rating”) was scored on a 7-point scale and then calculated as a percentage, which was the same method used to calculate domain scores, as described in previous studies (15, 29). For the second global rating item, a CPG was rated as high quality when the score of three domains considered as the most important was ≥50% of the maximum possible score, consistent with previous studies (15, 30, 31). The three domains were as follows: stakeholder involvement (domain 2), rigor of development (domain 3), and editorial independence (domain 6).

Before the assessment, researchers received online training using the AGREE II Online Training Tool. Then, a meeting was held to discuss the specific assessment criteria of AGREE II, and researchers assessed some CPGs of different levels and discussed the results with each other. The formal assessment was performed only when the intraclass correlation coefficient (ICC) was >0.8. During the assessment process, researchers carefully read the document of each CPG and its accompanying documents or information on the Internet to make an accurate judgment. If the researchers’ score on an item differed significantly (more than 2 points), a consensus was reached through discussion.

### 2.5. Statistical analysis

The researchers’ AGREE score was entered into Microsoft Excel (Microsoft, WA, United States), and the standardized score of each domain and the overall score of each CPG were calculated. Continuous variables were expressed as mean ± standard deviation (SD) (normal distribution) or median (Q1–Q3) (skewed distribution), and categorical variables were expressed as frequencies and percentages. The independent-sample t test/nonparametric tests (Kruskal-Wallis test)/chi-square tests/Fisher exact tests were used to compare the differences between the two groups. As the overall scores of AGREE II domain, overall rating, and item of included CPGs had both normal and nonnormal distributions, consistent with the previous studies (27), the mean (SD) and median (Q1–Q3) of overall scores were both presented for the convenience of observation and comparison. The number of each level of evidence and the strength of recommendation of each CPG were evaluated. The univariate linear regression model and the logistic regression model were used to assess the associations between the characteristics and each AGREE II domain score and overall assessment of included CPGs. An ICC with a 95% confidence interval (CI) with a two-way random-effects model was used to detect the inter-rater agreement to ensure that researchers’ understandings of each item were basically the same, and ICCs for each domain and overall rating scores were calculated. Consistent with previous studies (25, 26) and according to Landis and Koch (32), the degree of agreement between 0.01 and 0.20 was considered minor, between 0.21 and 0.40 as fair, between 0.41 and 0.60 as moderate, between 0.61 and 0.80 as substantial, and between 0.81 and 1.00 as very good.

In the sensitivity analysis, a series of analyses were performed to test the robustness of our findings. First, other criteria were used for the overall assessment to identify whether the overall rating score and the number of high-quality CPGs differed from the initial results. For the first global rating item (“overall rating”), the score of each CPG was based on the average score of the six domains, consistent with previous studies (27, 33). A stricter standard was used for the second global rating item, consistent with previous studies (27, 34). A CPG was classified as of high quality if the score of domain 3 (rigor of development) was >70% and the scores of all other domains and overall rating were > 50%. Second, the univariate regression analysis, restricted to CPGs published after 2015 or evidence based or MND as the target disease, was performed separately. This helped assess the association between characteristics and each AGREE II domain score and overall assessment of included CPGs, determine whether these associations were consistent across different types of CPGs, and reduce confounders.

All analyses were performed using the following software packages: R (http://www.R-project.org, The R Foundation), EmpowerStats (http://www.empowerstats.com, X&Y Solutions, Inc., MA, United States), and SPSS 23.0 (IBM, IL, United States). A *p* value less than 0.05 (two-sided) indicated a statistically significant difference.

## 3. Results

### 3.1. Study selection

**Figure 1:**
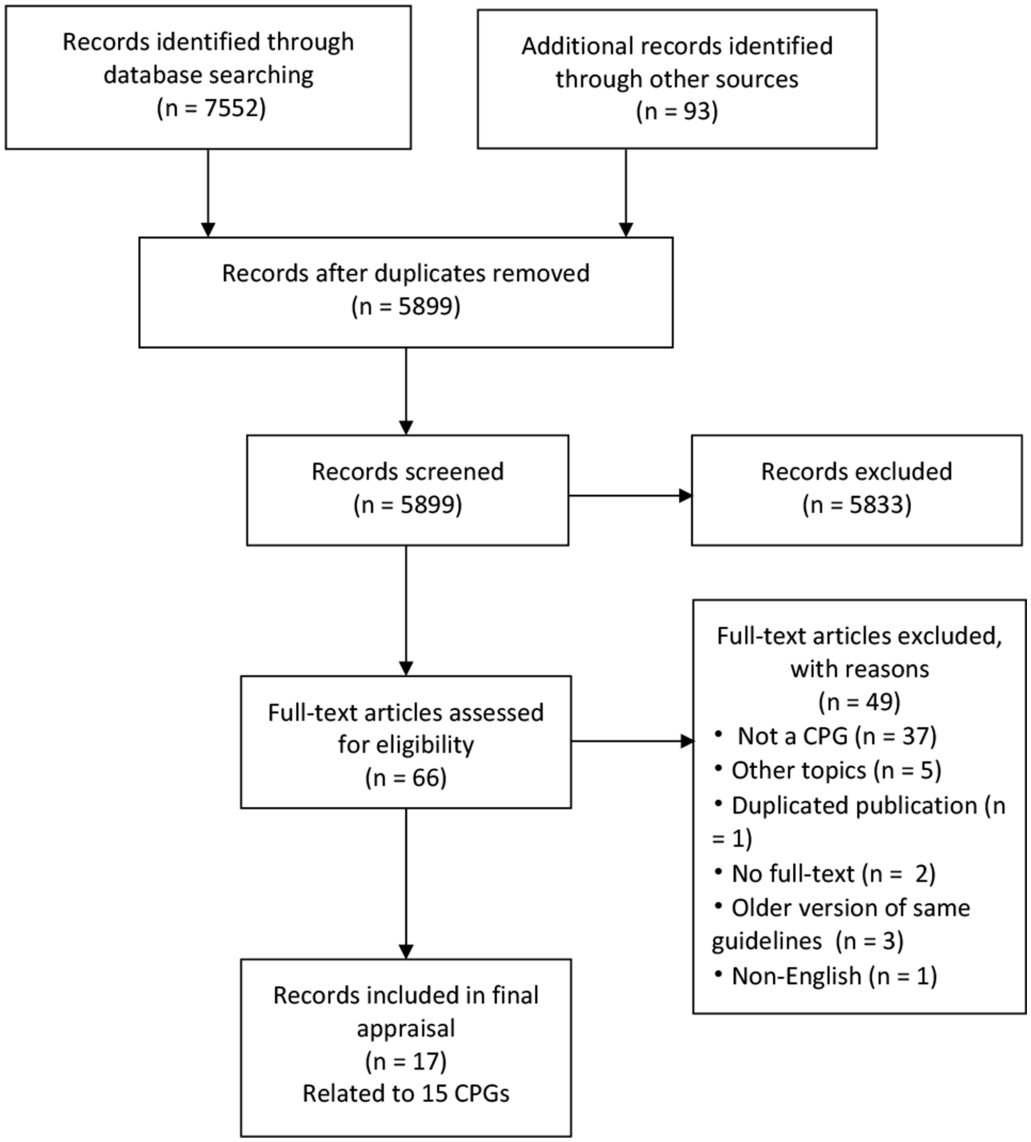
PRISMA flow diagram. CPG, Clinical practice guideline; PRISMA, Preferred Reporting Items for Systematic Reviews and Meta-Analyses.

A total of 5,899 records were yielded initially, and 15 CPGs were finally included for assessment after screening by title, abstract, and full text (Figure 1). Two CPGs were both published in two parts but considered as one (6, 35–37).

### 3.2. Characteristics of CPGs

The characteristics of included CPGs are displayed in Table 1 and Supplementary Table S1. Supplementary Table S2 shows the results of descriptive statistics of these characteristics. Nine CPGs were published after 2015. Eight CPGs were developed by a medical society and five by an expert panel. Ten CPGs were developed for MND and five for SMA; however, no CPG existed for PPMA. Five CPGs were originally from individual European countries, five were from individual North American countries, and the remaining were international, Europe, and Brazil. Six CPGs were updated. Three CPGs were for adults, one for children, and five for all ages; the rest did not specify the age group. Eight CPGs were considered evidence based and used a grading system. Seven CPGs reported the search dates. Only three CPGs used the CPG quality tool, such as the AGREE II tool. One CPG reported the inclusion of methodologists in the development team. Five CPGs were funded, four were not, and the remainder did not report the funding status. Compared with SMA CPGs, MND CPGs were significantly more likely to state the search dates (70.0% vs. 0.0%, *p* = 0.026) (Supplementary Table S3).

**Table 1:** Characteristics of included CPGs.

CPG ID (development organization^*^, year)	Disease	Type of development organization	Country/region of origin	Version	Age range of target population (adult/children/all ages)	Development method	Search dates covered	Used CPG quality tool	Included CPG methodologist
Pitarch Castellano et al. (44)	SMA	Expert panel	Spain	First	All ages	CB	Not stated	No	No
Neuromuscular Disorders Task Force，2022 (38)	MND	Expert panel	France	Updated	Not stated	CB	Since 2006 (end date not stated)	No	No
Shoesmith et al. (3)	MND	Expert panel	Canada	First	All ages	EB	2007–December 2018	Yes – GIN–McMaster Guideline Development Checklist	No
FILSLAN，2020 (39)	MND	Government	France	First	Not stated	CB	Not stated	No	Not stated
CTS, 2019 (65)	MND	Medical society	Canada	Updated	Adult	EB	Through to March 2018	Yes – AGREE II	No
NEALS bulbar subcommittee, 2019 (40)	MND	Medical society	USA	First	Not stated	CB	Not stated	No	No
BMA，2018 (42)	SMA	Medical society	Brazil	First	Children	EB	Not stated	No	Not stated
SMA Care Group，2018 (6, 35)	SMA	Expert panel	International	Updated	All ages	EB	Not stated	No	No
NICE，2019 (41)	MND	Government	UK	Updated	Adult	EB	Not stated	Yes – AGREE II	Yes
CORNEMUS, 2012 (43)	SMA	Expert panel	France	First	All ages	CB	Not stated	No	No
EFNS, 2012 (45)	MND	Medical society	Europe	Updated	Not stated	EB	2008–February 2011	No	Not stated
EFNS, 2010 (46)	MND	Medical society	Europe	First	Adult	EB	January 1965 –July 2009	No	Not stated
AAN，2009 (36, 37)	MND	Medical society	USA	Updated	Not stated	EB	1998–September 2007	No	Not stated
ACOG，2009 (47)	SMA	Medical society	USA	First	All ages	CB	Not stated	No	Not stated
IFCN, 2008 (50)	MND	Medical society	International	First	Not stated	CB	Not stated	No	Not stated

^*^First author given where there is no stated organisation; CPG, clinical practice guideline; EB, evidence-based; CB, consensus-based; FILSLAN, Filie` re Nationale de Sante´ SLA et autres Maladies rares du Neurone Moteur (National Heathcare Network for ALS and other rare motor neuron diseases); GIN, Guideline International Network; CTS, Canadian Thoracic society; AGREE, Appraisal of Guidelines for Research and Evaluation; NEALS, Northeast ALS; USA, United States of America; UK, United Kingdom; MND, motor neuron disease; SMA: spinal muscular atrophy; NICE, National Institute for Health and Clinical Excellence; NA, not applicable; CORNEMUS, network of French centers of expertise for neuromuscular diseases; EFNS, European Federation of Neurological Societies; AAN, American Academy of Neurology; ACOG, The American College of Obstetricians and Gynecologists; IFCN, International Federation of Clinical Neurophysiolog; BMA, Brazilian Medical Association.

### 3.3. Quality assessment of CPGs

Table 2 displays the overall mean (SD) and median (Q1–Q3) scores for each AGREE II domain, item, and overall rating of included CPGs. The mean overall rating score for all CPGs was 44.5% [SD = 24.7%, median = 39.0% (Q1–Q3, 25.0–58.5%)]. Overall, the highest domain score was for clarity of presentation (domain 4) [mean = 80.3% (SD, 14.8%), median = 85.0% (Q1–Q3, 67.0–92.0%)]. The next highest score was for scope and purpose (domain 1) [mean = 68.1% (SD, 23.8%), median = 67.0% (Q1–Q3, 52.0–88.5%)]. The lowest domain score was for applicability (domain 5) [mean = 24.4% (SD, 24.2%), median = 15.0% (Q1–Q3, 10.5–27.0%)], rigor of development (domain 3) [mean = 39.9% (SD, 29.5%), median = 28.0% (Q1–Q3, 14.0–57.5%)], and stakeholder involvement (domain 2) [mean = 40.3% (SD, 22.2%), median = 37.0% (Q1–Q3, 26.0–51.0%)]. The moderate domain score was for editorial independence (domain 6) [mean = 55.9% (SD, 34.4%), median = 47.0% (Q1–Q3, 32.0–94.0%)]. Compared with SMA CPGs, MND CPGs had a significantly lower domain 6 score [median = 80.5% (Q1–Q3, 47.8–96.2%) vs. median = 42.0% (Q1–Q3, 17.0–47.0%), *p* = 0.036] (Supplementary Table S4).

**Table 2:** Overall mean (SD) and median (Q1–Q3) scores for each AGREE II domain, item, and overall rating of included CPGs.

AGREE II domain, item and overall rating	Mean(SD) Median (Q1-Q3)
Domain 1: scope and purpose (%)	68.1 (23.8) 67.0 (52.0–88.5)
1. The overall objective(s) of the guideline is (are) specifically described.	6.1 (1.6) 7.0 (6.0–7.0)
2. The health question(s) covered by the guideline is (are) specifically described.	4.5 (2.2) 4.0 (3.0–7.0)
3. The population (patients, public, etc.) to whom the guideline is meant to apply is specifically described.	4.7 (1.7) 4.0 (3.0–7.0)
Domain 2: stakeholder involvement (%)	40.3 (22.2) 37.0 (26.0–51.0)
4. The guideline development group includes individuals from all relevant professional groups.	4.1 (1.8) 4.0 (3.0–6.0)
5. The views and preferences of the target population (patients, public, etc.) have been sought.	2.5 (1.8) 2.0 (1.0–4.0)
6. The target users of the guideline are clearly defined.	3.7 (2.4) 3.0 (2.0–6.0)
Domain 3: rigour of development (%)	39.9 (29.5) 28.0 (14.0–57.5)
7. Systematic methods were used to search for evidence.	3.4 (2.5) 4.0 (1.0–6.0)
8. The criteria for selecting the evidence are clearly described.	2.6 (2.2) 1.0 (1.0–4.0)
9. The strengths and limitations of the body of evidence are clearly described.	3.6 (2.6) 3.0 (1.0–6.0)
10. The methods for formulating the recommendations are clearly described.	3.4 (1.7) 4.0 (2.0–5.0)
11. The health benefits, side effects, and risks have been considered in formulating the recommendations.	4.9 (1.9) 5.0 (3.0–7.0)
12. There is an explicit link between the recommendations and the supporting evidence.	4.8 (2.1) 6.0 (3.0–6.0)
13. The guideline has been externally reviewed by experts prior to its publication.	2.1 (2.1) 1.0 (1.0–1.0)
14. A procedure for updating the guideline is provided.	2.3 (2.3) 1.0 (1.0–2.0)
Domain 4: clarity of presentation (%)	80.3 (14.8) 85.0 (67.0–92.0)
15. The recommendations are specific and unambiguous.	6.1 (0.9) 6.0 (6.0–7.0)
16. The different options for management of the condition or health issue are clearly presented.	5.8 (1.2) 6.0 (5.0–7.0)
17. Key recommendations are easily identifiable.	5.5 (2.0) 7.0 (4.0–7.0)
Domain 5: applicability (%)	24.4 (24.2) 15.0 (10.5–27.0)
18. The guideline describes facilitators and barriers to its application.	3.3 (1.8) 3.0 (2.0–5.0)
19. The guideline provides advice and/or tools on how the recommendations can be put into practice.	2.1 (2.0) 1.0 (1.0–2.0)
20. The potential resource implications of applying the recommendations have been considered.	2.4 (1.5) 2.0 (1.0–3.0)
21. The guideline presents monitoring and/or auditing criteria.	2.1 (1.8) 1.0 (1.0–2.0)
Domain 6: editorial independence (%)	55.9 (34.4) 47.0 (32.0–94.0)
22. The views of the funding body have not influenced the content of the guideline.	3.6 (2.6) 3.0 (1.0–7.0)
23. Competing interests of guideline development group members have been recorded and addressed.	5.1 (2.5) 6.0 (2.0–7.0)
Overall rating (%)	44.5 (24.7) 39.0 (25.0–58.5)

CPG, clinical practice guideline; AGREE, Appraisal of Guidelines for Research and Evaluation.

Item 1 [mean = 6.1 (SD, 1.6), median = 7.0 (Q1–Q3, 6.0–7.0)] and item 15 [mean = 6.1 (SD, 0.9), median = 6.0 (Q1–Q3, 6.0–7.0)] had the highest scores. Item 21 [mean = 2.1 (SD, 1.8), median = 1.0 (Q1–Q3, 1.0–2.0)], item 19 [mean = 2.1 (SD, 2.0), median = 1.0 (Q1–Q3, 1.0–2.0)], and item 13 [mean = 2.1 (SD, 2.1), median = 1.0 (Q1–Q3, 1.0–1.0)] had the lowest scores.

**Figure 2:**
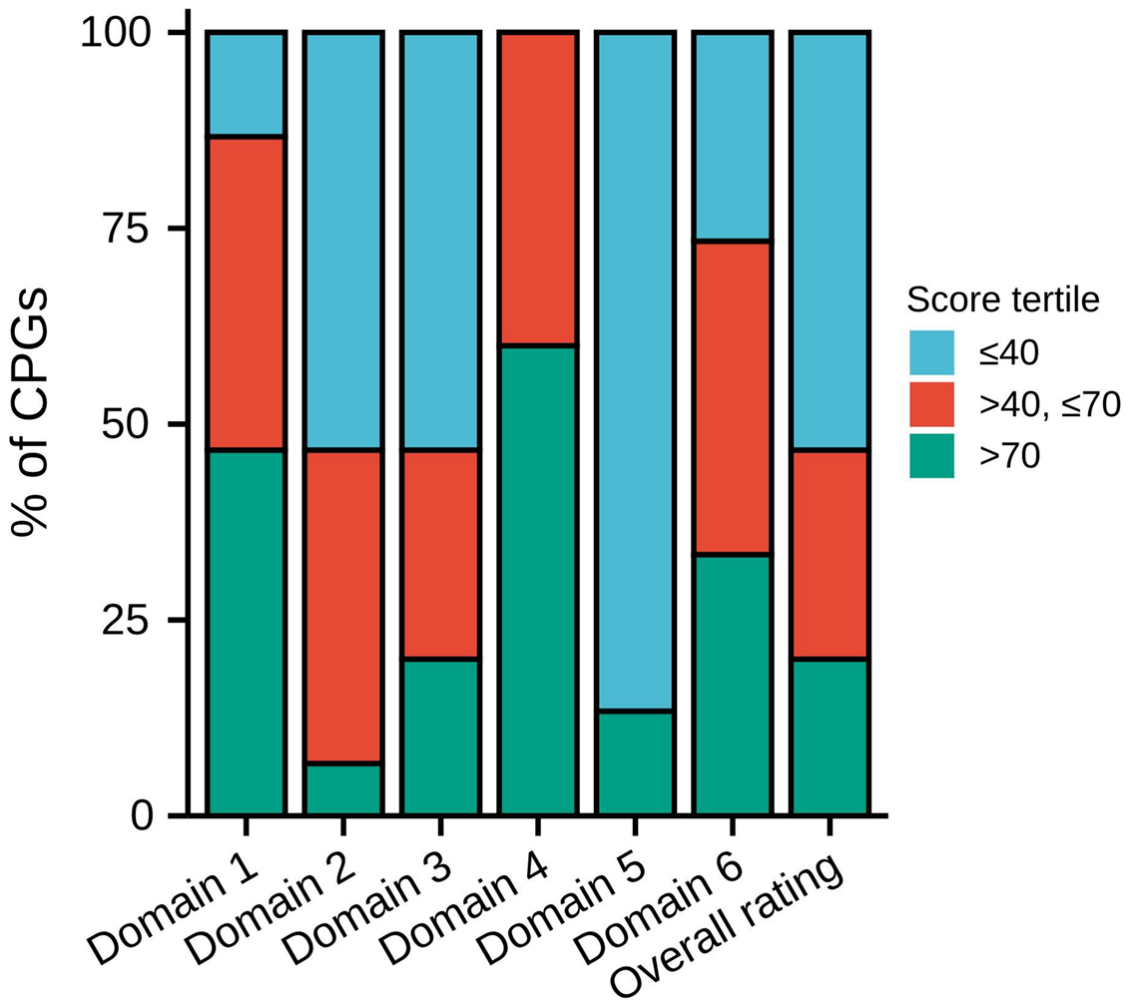
Distribution of the degree of score across each domain and overall rating. CPG, Clinical practice guideline.

Table 3 displays the AGREE II domain scores and overall assessment of each CPG. Figure 2 displays the distribution of the degree of score across each domain and the overall rating. In the domain scope and purpose, seven CPGs received high ratings, which were scored >70%. Six CPGs received moderate ratings, which were scored >40% and ≤ 70%. The National Institute for Health and Clinical Excellence (NICE) CPG received the highest scores (100%), and the Canadian Thoracic Society (CTS), Brazilian Medical Association, and the 2020 Canada CPG also got high scores, which were all >90%. The distribution of the rating degree was similar in the stakeholder involvement and rigor of development domains, with eight CPGs receiving low ratings, which were ≤ 40%. The 2020 Canada CPG received the highest scores in these two domains (domain 2: 89%, domain 3: 91%). In addition, the NICE CPG also received high ratings in both domains (domain 2: 70%, domain 3: 76%). The CTS received the second highest score in domain 3 (90%). Regarding the domain clarity of the presentation, the results were satisfactory. More than half of the CPGs received high ratings, and none received low ratings. In the domain applicability, thirteen CPGs received low ratings and only two CPGs received high ratings, and these two CPGs were the CTS (85%) and NICE CPG (74%). In the editorial independence domain, five CPGs received high ratings (33.3%) and six received moderate ratings (40.0%). Many CPGs lacked funding information and statements of competing interests. In the overall rating, eight CPGs received low ratings, and only the CTS (89%), NICE (78%), and 2020 Canada CPG (83%) received high ratings.

**Table 3:** AGREE II domain scores and overall assessment of included CPGs.

CPG ID	Domain 1: scope and purpose (%)	Domain 2: stakeholder involvement (%)	Domain 3: rigour of development (%)	Domain 4: clarity of presentation (%)	Domain 5: applicability (%)	Domain 6: editorial independence (%)	Overall rating (%)	Quality (high/low)
Pitarch Castellano et al. (44)	37	56	15	85	22	42	28	Low
Neuromuscular Disorders Task Force, 2022 (38)	56	33	26	67	28	100	39	Low
Shoesmith et al. (3)	94	89	91	93	35	100	83	High
FILSLAN，2020 (39)	46	9	3	69	4	47	11	Low
CTS, 2019 (65)	96	63	90	91	85	94	89	High
NEALS bulbar subcommittee, 2019 (40)	81	26	27	67	19	22	28	Low
BMA，2018 (42)	96	26	66	63	10	47	44	Low
SMA Care Group，2018 (6, 35)	67	46	28	67	15	17	39	Low
NICE，2019 (41)	100	70	76	96	74	67	78	High
CORNEMUS, 2012 (43)	61	15	13	52	13	47	22	Low
EFNS, 2012 (45)	61	46	45	98	26	94	56	Low
EFNS, 2010 (46)	83	43	47	89	1	50	50	Low
AAN，2009 (36, 37)	74	37	49	96	8	97	61	Low
ACOG，2009 (47)	22	28	9	91	11	0	22	Low
IFCN, 2008 (50)	48	17	13	80	15	14	17	Low

AGREE, Appraisal of Guidelines for Research and Evaluation; CPG, clinical practice guideline; FILSLAN, Filie` re Nationale de Sante´ SLA et autres Maladies rares du Neurone Moteur (National Heathcare Network for ALS and other rare motor neuron diseases); CTS, Canadian Thoracic society; NEALS, Northeast ALS; SMA, spinal muscular atrophy; NICE, National Institute for Health and Clinical Excellence; CORNEMUS, network of French centers of expertise for neuromuscular diseases; EFNS, European Federation of Neurological Societies; AAN, American Academy of Neurology; ACOG, The American College of Obstetricians and Gynecologists; IFCN, International Federation of Clinical Neurophysiolog; BMA, Brazilian Medical Association.

Among the 15 CPGs, 3 were of high quality, which was consistent with the result of the overall rating. These three CPGs were all for MND, while the NICE and CTS CPGs were for adults, and the 2020 Canada CPG was for all ages. The CTS and 2020 Canada CPGs were from Canada, and the NICE CPG was from the UK. All three CPGs were evidence based and used the CPG quality tool.

The inter-rater reliability was very good for all domains and the overall rating (Table 4). Supplementary File 2 displays the individual scoring of the AGREE II tool for each CPG.

**Table 4:** Inter-rater reliability for AGREE II domain and overall rating.

AGREE II domain and overall rating	ICC (95%CI)
Domain 1: scope and purpose	0.916 (0.866, 0.950)
Domain 2: stakeholder involvement	0.913 (0.863, 0.948)
Domain 3: rigour of development	0.950 (0.933, 0.963)
Domain 4: clarity of presentation	0.813 (0.716, 0.885)
Domain 5: applicability	0.897 (0.848, 0.934)
Domain 6: editorial independence	0.974 (0.953, 0.987)
Overall rating	0.887 (0.758, 0.957)

AGREE, Appraisal of Guidelines for Research and Evaluation; ICC, intraclass correlation coefficients; CI, confidence interval.

### 3.4. Level of evidence and strength of recommendation of CPGs

Among the 15 CPGs, 8 were evidence based. Table 5 displays the grading systems used and the distribution of the level of evidence and strength of recommendation among these CPGs. Two CPGs used the adapted Grading of Recommendations Assessment, Development and Evaluation (GRADE) system, one used the combined Oxford Centre for Evidence-based Medicine and GRADE system, one used the American Academy of Pediatrics criteria, two used the adapted American Academy of Neurology (AAN) criteria, one used the AAN criteria, and one did not report the name of the grading system. Four CPGs graded evidence by a body of evidence, whereas four CPGs graded only individual studies. One CPG did not grade the strength of recommendations, and two CPG did not report the strength of specific recommendations.

**Table 5:** Grading systems used and the distribution of the level of evidence and strength of recommendation among included evidence-based CPGs.

CPG ID	Name of grading system	Level of evidence, No. (%)	Strength of recommendation, No. (%)
Shoesmith et al. (3)	Not stated	A: 2 (2.3) B: 21 (23.9) C: 16 (18.2) EC: 49 (55.7)	No
CTS, 2019 (65)	Adapted GRADE system	A: 1 (4.5) B: 7 (31.8) C: 14 (63.6)	1: 17 (77.3) 2: 5 (22.7)
BMA，2018 (42)	Combined OCEBM and GRADE system	A: 2 (100) B: 0 D: 0	Strong Moderate Weak Very weak (The strength of specific recommendations was not stated)
SMA Care Group，2018 (6, 35)	AAP criteria	A: 3 (2.8) B: 2 (1.9) C: 21 (19.6) D: 81 (75.7)	Strong recommendation Recommendation Option No recommendation (The strength of specific recommendations was not stated)
NICE，2019 (41)	Adapted GRADE system	High: 0 Moderate: 5 (3.5) Low: 18 (12.6) Very low: 120 (83.9)	Recommendations that must or must not be used: 0 Recommendations that should or should not be offered: 105 (85.4) Recommendations that could be offered: 18 (14.6)
EFNS, 2012 (45)	Adapted AAN criteria	I: 13 (8.1) II: 10 (6.2) III: 19 (11.8) IV: 119 (73.9)	A: 3 (2.9) B: 4 (3.9) C: 4 (3.9) GCPP: 91 (89.2)
EFNS, 2010 (46)	Adapted AAN criteria	I: 0 II: 0 III: 0 IV: 11 (100)	A: 0 B: 0 GCPP: 11 (100)
AAN，2009 (36, 37)	AAN criteria	I: 11 (11.3) II: 13 (13.4) III: 73 (75.3) IV: 0	A: 2 (5.1) B: 9 (23.1) C: 10 (25.6) U: 18 (46.2)

CPG, clinical practice guideline; EC, Expert consensus; CTS, Canadian Thoracic society; GRADE, Grading of Recommendations Assessment, Development and Evaluation; OCEBM, Oxford Centre for Evidence-based Medicine; SMA, spinal muscular atrophy; AAP, American Academy of Pediatrics; NICE, National Institute for Health and Clinical Excellence; EFNS, European Federation of Neurological Societies; AAN, American Academy of Neurology; GCPP, Good Clinical Practice Points; BMA, Brazilian Medical Association.

Although the CPGs used different grading systems, their grading criteria for the level of evidence and strength of recommendations were similar. Most of the evidence for which CPGs graded specific evidence (85.7%) were C, low, D, very low, EC, III, and IV level, indicating low-quality evidence. In the same way, most of the recommendations for which CPGs graded specific recommendations (88.2%) were graded as C, GCPP, U, 2, and weak strength, indicating weak recommendations.

### 3.5. Relationship between characteristics and AGREE II domain score and overall assessment of CPGs

Table 6 displays the results of the relationship between characteristics and AGREE II domain score and overall assessment of included CPGs. Compared with the MND CPGs, SMA CPGs had a lower domain 6 score (β = -37.9, 95% CI: −70.3 to −5.5). Compared with the CPGs of the first version, the updated CPGs had a higher domain 5 score (β = 24.9, 95% CI: 2.7–47.1), domain 6 score (β = 37.2, 95% CI: 6.3–68.1), and overall rating score (β = 26.4, 95% CI: 4.3–48.6). Compared with the CPGs for adults, CPGs for all ages had a lower domain 1 score (β = −36.8, 95% CI: −64.9 to −8.7), domain 3 score (β = −39.8, 95% CI: −76.5 to −3.1), and domain 5 score (β = −34.1, 95% CI: −64.7 to −3.6). CPGs that did not state the age range of the target population had a lower domain 1 score (β = −32.0, 95% CI: −59.3 to −4.7), domain 2 score (β = −30.7, 95% CI: −58.8 to −2.5), domain 3 score (β = −43.8, 95% CI: −79.3 to −8.3), domain 5 score (β = −36.7, 95% CI: −66.2 to −7.1), and overall rating score (β = -37.0, 95% CI: −68.1 to −5.9).

**Table 6:** Relationship between characteristics and AGREE II domain score and overall assessment of included CPGs.

Characteristic	Statistics	Domain 1, % (β, 95%CI, *p*-value)	Domain 2, % (β, 95%CI, *p*-value)		Domain 3, % (β, 95%CI, *p*-value)	Domain 4, % (β, 95%CI, *p*-value)	Domain 5, % (β, 95%CI, *p*-value)	Domain 6, % (β, 95%CI, *p*-value)	Overall rating, % (β, 95%CI, *p*-value)	Quality (OR, 95%CI, *p*-value)
Disease										
MND	10 (66.7%)	Reference	Reference		Reference	Reference	Reference	Reference	Reference	Reference
SMA	5 (33.3%)	−17.3 (−42.0, 7.4) 0.194	−9.1 (−33.3, 15.1) 0.474		−20.5 (−51.4, 10.4) 0.216	−13.0 (−27.9, 1.9) 0.110	−15.3 (−41.0, 10.4) 0.264	−37.9 (−70.3, −5.5) 0.039	−20.2 (−45.4, 5.0) 0.140	–^*^
Year										
≤ 2015	6 (40.0%)	Reference	Reference		Reference	Reference	Reference	Reference	Reference	Reference
> 2015	9 (60.0%)	16.6 (−7.2, 40.4) 0.195	15.4 (−6.8, 37.7) 0.196		17.6 (−12.5, 47.7) 0.274	−6.8 (−22.2, 8.6) 0.404	20.1 (−3.5, 43.7) 0.118	9.2 (−27.4, 45.8) 0.630	10.8 (−15.0, 36.6) 0.427	–^*^
Type of development organization										
Expert panel	5 (33.3%)	Reference	Reference		Reference	Reference	Reference	Reference	Reference	Reference
Government	2 (13.3%)	10.0 (−31.5, 51.5) 0.645	−8.3 (−46.3, 29.7) 0.676		4.9 (−46.8, 56.6) 0.856	9.7 (−14.6, 34.0) 0.449	16.4 (−25.2, 58.0) 0.455	−4.2 (−64.8, 56.4) 0.894	2.3 (−41.3, 45.9) 0.919	4.0 (0.1, 137.0) 0.442
Medical society	8 (53.3%)	7.1 (−21.2, 35.4) 0.630	−12.1 (−37.9, 13.8) 0.379		8.7 (−26.6, 43.9) 0.639	11.6 (−5.0, 28.1) 0.195	−0.7 (−29.1, 27.6) 0.961	−8.9 (−50.2, 32.3) 0.678	3.7 (−26.0, 33.4) 0.812	0.6 (0.0, 11.8) 0.718
Country/region of origin										
European country	5 (33.3%)	Reference	Reference		Reference	Reference	Reference	Reference	Reference	Reference
North American country	5 (33.3%)	13.4 (−17.4, 44.2) 0.410	12.0 (−16.5, 40.5) 0.426		26.6 (−9.9, 63.1) 0.178	13.8 (−4.3, 31.9) 0.162	3.4 (−27.1, 33.9) 0.831	2.0 (−42.7, 46.7) 0.932	21.0 (−9.6, 51.6) 0.204	2.7 (0.2, 45.1) 0.497
Other	5 (33.3%)	11.0 (−19.8, 41.8) 0.497	−1.0 (−29.5, 27.5) 0.946		13.2 (−23.3, 49.7) 0.492	5.6 (−12.5, 23.7) 0.557	−14.8 (−45.3, 15.7) 0.361	−16.2 (−60.9, 28.5) 0.491	5.6 (−25.0, 36.2) 0.726	–^*^
Version										
First	9 (60.0%)	Reference	Reference		Reference	Reference	Reference	Reference	Reference	Reference
Updated	6 (40.0%)	12.6 (−12.0, 37.1) 0.334	14.8 (−7.5, 37.2) 0.216		20.8 (−8.7, 50.3) 0.191	9.3 (−5.7, 24.3) 0.247	24.9 (2.7, 47.1) 0.047	37.2 (6.3, 68.1) 0.035	26.4 (4.3, 48.6) 0.036	4.0 (0.3, 58.6) 0.311
Age range of target population										
Adult	3 (20.0%)	Reference	Reference		Reference	Reference	Reference	Reference	Reference	Reference
Children	1 (6.7%)	3.0 (−41.5, 47.5) 0.897	−32.7 (−78.6, 13.3) 0.191		−5.0 (−63.0, 53.0) 0.869	−29.0 (−61.7, 3.7) 0.110	−43.3 (−91.6, 5.0) 0.106	−23.3 (−105.7, 59.1) 0.590	−28.3 (−79.1, 22.4) 0.297	–^*^
All ages	5 (33.3%)	−36.8 (−64.9, −8.7) 0.026	−11.9 (−40.9, 17.2) 0.441		−39.8 (−76.5, −3.1) 0.057	−14.4 (−35.1, 6.3) 0.200	−34.1 (−64.7, −3.6) 0.051	−29.1 (−81.2, 23.0) 0.297	−33.5 (−65.6, −1.5) 0.065	0.1 (0.0, 3.2) 0.210
Not stated	6 (40.0%)	−32.0 (−59.3, −4.7) 0.042	−30.7 (−58.8, −2.5) 0.056		−43.8 (−79.3, −8.3) 0.034	−12.5 (−32.6, 7.6) 0.247	−36.7 (−66.2, −7.1) 0.033	−8.0 (−58.5, 42.5) 0.762	−37.0 (−68.1, −5.9) 0.040	–^*^
Development method										
CB	7 (46.7%)	Reference	Reference		Reference	Reference	Reference	Reference	Reference	Reference
EB	8 (53.3%)	33.7 (16.7, 50.7) 0.002	26.2 (7.7, 44.7) 0.016		46.4 (28.3, 64.4) <0.001	13.6 (−0.0, 27.3) 0.073	15.8 (−8.3, 39.8) 0.221	31.9 (0.0, 63.7) 0.071	38.6 (23.4, 53.9) <0.001	–^*^
Stated search dates										
Yes	7 (46.7%)	Reference	Reference		Reference	Reference	Reference	Reference	Reference	Reference
No	8 (53.3%)	−9.4 (−33.9, 15.1) 0.465	−12.4 (−34.7, 10.0) 0.298		−21.9 (−50.6, 6.7) 0.157	−14.0 (−27.5, −0.4) 0.065	−7.3 (−32.5, 17.9) 0.581	−42.3 (−70.3, −14.3) 0.011	−22.4 (−45.4, 0.5) 0.077	0.4 (0.0, 5.1) 0.448
Used CPG quality tool										
Yes	3 (20.0%)	Reference	Reference		Reference	Reference	Reference	Reference	Reference	Reference
No	12 (80.0%)	−35.7 (−60.1, −11.2) 0.013	−42.2 (−60.1, −24.2) <0.001		−57.2 (−80.2, −34.3) <0.001	−16.3 (−33.6, 0.9) 0.086	−50.3 (−66.6, −34.1) <0.001	−38.9 (−78.9, 1.1) 0.079	−48.6 (−67.3, −29.8) <0.001	–^*^
Included CPG methodologist										
Yes	1 (6.7%)	Reference	Reference		Reference	Reference	Reference	Reference	Reference	Reference
No	7 (46.7%)	−29.7 (−78.6, 19.2) 0.257	−23.1 (−65.3, 19.0) 0.303		−34.6 (−96.6, 27.4) 0.296	−21.4 (−51.6, 8.8) 0.190	−43.0 (−81.9, −4.1) 0.051	−6.7 (−83.5, 70.0) 0.867	−31.1 (−81.7, 19.4) 0.251	–^*^
Not stated	7 (46.7%)	−38.6 (−87.5, 10.3) 0.148	−40.6 (−82.8, 1.6) 0.084		−42.9 (−104.9, 19.2) 0.201	−12.3 (−42.5, 17.9) 0.441	−63.3 (−102.2, −24.3) 0.008	−17.1 (−93.9, 59.6) 0.669	−40.7 (−91.3, 9.9) 0.141	–^*^
Used grading system										
Yes	8 (53.3%)	Reference	Reference		Reference	Reference	Reference	Reference	Reference	Reference
No	7 (46.7%)	−33.7 (−50.7, −16.7) 0.002	−26.2 (−44.7, −7.7) 0.016		−46.4 (−64.4, −28.3) <0.001	−13.6 (−27.3, 0.0) 0.073	−15.8 (−39.8, 8.3) 0.221	−31.9 (−63.7, −0.0) 0.071	−38.6 (−53.9, −23.4) <0.001	–^*^
With funding sources										
Yes	5 (33.3%)	Reference	Reference	Reference	Reference	Reference	Reference	Reference		Reference
No	4 (26.7%)	−6.3 (−37.3, 24.8) 0.700	−4.8 (−33.4, 23.7) 0.745	5.5 (−32.5, 43.5) 0.781	7.4 (−12.1, 26.9) 0.471	5.2 (−24.6, 34.9) 0.740	52.3 (19.1, 85.4) 0.009	12.3 (−17.0, 41.5) 0.428		0.5 (0.0, 9.0) 0.638
Not stated	6 (40.0%)	−20.5 (−48.5, 7.5) 0.177	−20.1 (−45.9, 5.7) 0.152	−21.5 (−55.8, 12.8) 0.243	−5.8 (−23.4, 11.8) 0.533	−21.4 (−48.3, 5.4) 0.144	−5.2 (−35.1, 24.8) 0.741	−19.5 (−45.9, 6.9) 0.173		–^*^

^*^The model failed because of the small sample size; AGREE, Appraisal of Guidelines for Research and Evaluation; CPG, clinical practice guideline; CI, confidence interval; OR, odds ratio; MND, motor neuron disease; SMA, spinal muscular atrophy; EB, evidence-based; CB, consensus-based.

Compared with the consensus-based CPGs, evidence-based CPGs had a higher domain 1 score (β = 33.7, 95% CI: 16.7–50.7), domain 2 score (β = 26.2, 95% CI: 7.7–44.7), domain 3 score (β = 46.4, 95% CI: 28.3–64.4), and overall rating score (β = 38.6, 95% CI: 23.4–53.9). Compared with CPGs that stated the search dates, those that did not state the search dates had a lower domain 6 score (β = −42.3, 95% CI: −70.3 to −14.3). Compared with the CPGs using the CPG quality tool, those not using the CPG quality tool had a lower domain 1 score (β = −35.7, 95% CI: 60.1 to −11.2), domain 2 score (β = −42.2, 95% CI: −60.1 to −24.2), domain 3 score (β = −57.2, 95% CI: −80.2 to −34.3), domain 5 score (β = −50.3, 95%CI: −66.6 to −34.1), and overall rating score (β = −48.6, 95% CI: −67.3 to −29.8). Compared with the CPGs including a CPG methodologist, those not including a CPG methodologist had a lower domain 5 score (β = −43.0, 95% CI: −81.9 to −4.1), and those not stating had a lower domain 5 score (β = −63.3, 95% CI: −102.2 to −24.3). A comparison between CPGs using and not using the grading system showed the same results as that between consensus-based and evidence-based CPGs.

We also found that the year of publication, type of development organization, country/region of origin, and funding sources were not associated with the AGREE II domain score and overall assessment of included CPGs.

### 3.6. Sensitivity analysis

Supplementary Table S5 displays the overall assessment of included CPGs using other criteria. The mean overall rating score for all CPGs was 51.5% [SD = 19.9%, median = 51.0% (Q1–Q3, 36.5–61.0%)]. For the second global rating item, only the CTS CPG and the NICE CPG were of high quality under the stricter evaluation criteria. The 2020 Canada CPG was rated as low quality because its domain 5 score was less than 50%. Supplementary Tables S6–S8, respectively, display the relationship between characteristics and AGREE II domain score and overall assessment of included CPGs published after 2015, evidence-based CPGs, and CPGs for MND. The relationship between characteristics and each AGREE II domain score and overall assessment of different types of CPGs were generally consistent with the main analyses that the results in all included CPGs.

## 4. Discussion

This study was novel in assessing the quality of CPG for MNDs or related disorders using the AGREE II instrument and identifying the distribution of the level of evidence among these CPGs. The quality of CPGs for MNDs or related disorders varied widely. The overall quality of these CPGs was generally poor, and only three CPGs were rated as high quality. However, in a more stringent assessment standard, only two CPGs were of high quality. In comparison, the highest domain score was for clarity of presentation (domain 4) and the lowest domain score was for applicability (domain 5). Eight CPGs were considered evidence based, and most of the evidence (85.7%) on which these CPGs’ recommendations were based was low-quality evidence. Despite the improvement in the quality of CPGs in recent years, contemporary CPGs for MNDs or related disorders still lacked a consolidated evidence basis to provide recommendations for clinical practice. We also identified some factors that could affect the quality of CPGs, which could also serve as aspects for improvement.

Consistent with other studies (15, 30, 31), we used a less-stringent criterion in the overall assessment to judge whether a CPG was of high or low quality, but most CPGs were of poor quality. The development of CPGs requires significant resource consumption. Spending resources on low-quality CPGs with ineffective care recommendations is a waste and confusing for users (15). Coexisting problems are the uneven geographical distribution of CPGs and the duplication of CPGs. Most CPGs were from Europe and North America, only two CPGs were international, and one was from Brazil. Only Canada and the United Kingdom had high-quality CPGs, while other countries lacked them. All CPGs were for MND and SMA, of which SMA CPGs were all of low quality. No CPGs existed for PPS, although the prospect for the future is a continuous and ever-increasing demand for rehabilitation programs and management of patients with PPS (48, 49); currently around 18 million people are still affected by paralytic poliomyelitis (11). Dedicating resources to develop fewer, higher quality, and less-“redundant” CPGs can help reduce inefficient resource usage and user confusion (15, 51). Cooperation between countries and associations should be strengthened to reduce overlapping efforts and focus efforts and resources on developing high-quality CPGs and areas that need to be addressed.

Another problem is the inconsistent terminology used by CPG developers to describe the MND condition for which the CPGs are used. Also CPGs for MND include some CPGs for “amyotrophic lateral sclerosis (ALS)” and some for “MND.” Terminology can be confusing. MND is mainly divided into four categories: ALS, progressive bulbar palsy, progressive amyotrophy, and primary lateral sclerosis. When patients present with both upper and lower motor neuron signs, the disease is referred to as ALS, which is the most common form of MND (52). However, in the United Kingdom and Australia, MND is used as a general term for these disorders and also refers to the ALS subtype; however, in the United States, ALS is more commonly used as a general term and also denotes the ALS subtype (52). Consistent terminology is needed to define MND conditions, regardless of developer/professional group, to reduce CPG duplication and user confusion.

Many aspects of CPG development need to be improved. The domains of AGREE II that these aspects relate to are stakeholder involvement (domain 2), rigor of development (domain 3), applicability (domain 5), and editorial independence (domain 6). CPGs having problems in these domains were consistent with other studies (15, 19, 51). Among these, the problems of poor applicability and editorial independence of CPGs always existed, although the overall quality of CPGs in different health fields improved (51).

Stakeholder involvement reflects how well the CPG represents the views of its intended users, including patients. During the CPG development process, patients’ views, preferences, and expectations regarding care have become increasingly important (53). However, the overall score was low, with 53.3% of CPG scores ≤40% and only one CPG scoring greater than 70%. Most CPGs do not provide details about the involvement of patients or their representatives. Patients are important stakeholders and should be involved in the development process of CPGs, although this may introduce patient biases about costs, cultural background, and expectations (54).

Rigor of development is the most critical domain, as it significantly affects confidence in implementing CPGs (55), while nonsystematic development tends to lead to poor-quality CPGs (56). The strong heterogeneity found among the scores of included CPGs (Table 2) attested to the existence of significant gaps in the methodological development of the CPGs. Seven CPGs were not considered evidence based and did not use the grading system, or describe literature search and selection methods. The low score might be due to insufficient methodological consultation (55) or unfamiliarity with CPG development standards, poor reporting (19), or poor performance of the external peer review and update process (53).

The domain applicability considers factors that may affect guideline implementation, including identifying facilitators and barriers, providing tools for applying recommendations, identifying potentially relevant resources, and auditing standards (24). Thirteen CPGs received low ratings scoring ≤40%. The low applicability significantly hindered the implementation of CPG recommendations. CPG developers should conduct pilot tests to ensure feasibility before publication (57). In addition, all CPGS were just published as articles in journals. New ways to increase user adoption and usability should be considered by CPG developers (15), such as the use of smartphone applications (58, 59) or digital CPG platforms (60).

Editorial independence is also an important domain of CPG quality (61), and with only two statements required, it should be easy to score high (15). However, the overall score in this domain was not high [mean = 55.9% (SD, 34.4%)], with only five CPGs receiving high ratings, scoring >70%. Six CPGs did not state funding sources. Conflicts of interest are a common source of bias (62) and are often underestimated (63). CPG developers should clearly report conflicts of interest, including rigorous review processes and transparent review rules (64).

Overall, the NICE, CTS, and 2020 Canada CPGs (3, 41, 65) had the highest overall rating scores and were also rated as high quality. Based on this, these three CPGs should be favored by clinicians and policy makers, and are worthy of application in clinical practice.

Besides focusing on improving the transparency and methodological rigor of the CPG development process, CPGs should rely more on the growing body of evidence (26). However, nearly half of the CPGs for MNDs or related disorders were not considered evidence based, and most of the recommendations (85.7%) in the evidence-based CPGs were based on low-quality evidence, which were largely from observational studies or expert consensus. This finding constituted an obstacle to establishing CPGs for MND or related disorders, as recommendations were based on low-quality evidence, and it also showed a gap between clinical practice evidence and current medical research. Further research is needed on managing MND or related disorders, and more evidence-based recommendations would be extremely important to improve the standard of care for patients with MND or related disorders. Moreover, given that certain clinical questions may not addressed by high-quality research, it is expert consensus that can fill these knowledge gaps. Despite expert consensus may lack support from high-quality evidence, they can still provide valuable information. Consequently, CPGs developers should pay more attention to the rigor and standardization of consensus method and explore how to guide users to adopt expert consensus accurately.

Some characteristics and factors were found to be associated with the quality of CPGs. Specifically, among the CPGs for MND or related disorders, CPGs that were for MND, stated search dates, and included CPG methodologist were associated with higher scores in some specific domains, whereas CPGs that were updated, for adults, and evidence based, and used CPG quality tool and a grading system were associated with higher scores in both some specific domains and overall rating. In addition, the three CPGs that used the CPG quality tools were all rated as high quality, two of which used the AGREE II instrument. Therefore, the use of CPG quality tools, especially the AGREE II instrument, needs to be emphasized and improved during the development of CPGs. In addition, attention should also be paid to the use of the grading system, which has the greatest impact on the domain 3 score. The GRADE system is used to assess the level of evidence, while the AGREE II instrument is used to guide the CPG development process and set reporting standards (27), and they complement each other. Moreover, methodologists should be brought into the development team and should pay attention to CPG development details, such as providing the search date range of literature evidence.

This study had several limitations. Firstly, the search might have missed some CPGs, although the authors systematically searched major scientific databases and online guideline repositories. Secondly, only CPGs published in English were included, which might have excluded high-quality non-English CPGs, resulting in a lack of representation of CPGs from less-developed countries. Thirdly, the assessment of CPGs might reflect the researcher’s perspective, although our research team was multidisciplinary (including neurologists and other specialists, methodologists and other researchers), these limitations were unavoidable. Fourth, the AGREE II scoring system relied on the understandability and comprehensiveness of CPGs’ reporting and did not reflect the quality and strength of evidence (29). However, this study additionally analyzed the distribution of the level of evidence among included CPGs. Fifth, the AGREE II instrument did not provide a clear cut-off point to distinguish between high-quality and low-quality CPGs. To this end, based on some previous studies, we used a more widely used and less-stringent method to distinguish between high-quality and low-quality CPGs as the main analysis. Also, we used a more sensitive and stringent method to distinguish between high-quality and low-quality CPGs as a sensitivity analysis. Sixth, due to the small sample size, only univariate analysis was used to explore the relationship between CPG characteristics and AGREE II scores. However, we tried to reduce confounders by limiting the analysis to CPGs with certain characteristics, as a sensitivity analysis.

## 5. Conclusion

The quality of CPGs for MNDs or related disorders varied widely, and the overall quality was poor. No CPG existed for PPMA. Most recommendations were based on low-quality evidence. Many areas still need improvement, especially in the domains of stakeholder involvement, rigor of development, and applicability. CPGs for MNDs or related disorders should formulate recommendations with high-quality evidence and should be developed through rigorous methodology and transparency to minimize bias from external sources, and CPG quality tools should be used. In addition, a significant amount of studies on MNDs are still needed to fill the large evidence gap in the CPGs for these diseases.

## Data availability statement

The original contributions presented in the study are included in the article/Supplementary material, further inquiries can be directed to the corresponding authors.

## Author contributions

J-YO, LW, L-ML, and H-FP: study conception or design. J-YO, J-JL, JX, J-YL, and YL: data acquisition. J-YO, J-JL, JX, and J-YL: data analysis. J-YO, J-JL, and JX: manuscript writing. LW: funding acquisition. J-YO, J-JL, JX, J-YL, YL, Y-ZL, L-ML, H-FP, and LW: revision of manuscript. All authors contributed to the article and approved the submitted version.

## Funding

This study was supported by the Youth Program of the National Natural Science Foundation of China (NO: 81904297), the Innovation Team and Talents Cultivation Program of National (No: ZYYCXTD-C-202004), the Special Project of “Lingnan Modernization of Traditional Chinese Medicine” in 2019 Guangdong Provincial R & D Program (NO: 2020B1111100008), the Key Laboratory Program of Universities in Guangdong Province (NO:2018KSYS006), and the Discipline Collaborative Innovation Team Program of Double First-class and High-level Universities for Guangzhou University of Chinese Medicine (NO: 2021XK01).

## Conflict of interest

The authors declare that the research was conducted in the absence of any commercial or financial relationships that could be construed as a potential conflict of interest.

## Publisher’s note

All claims expressed in this article are solely those of the authors and do not necessarily represent those of their affiliated organizations, or those of the publisher, the editors and the reviewers. Any product that may be evaluated in this article, or claim that may be made by its manufacturer, is not guaranteed or endorsed by the publisher.
