# Supplementary material for: Quality appraisal of clinical practice guidelines for motor neuron diseases or related disorders using the AGREE II instrument
Source: Front Neurol. 2023 Jul 17;14:1180218. doi: 10.3389/fneur.2023.1180218 (PMC10388716; doi:10.3389/fneur.2023.1180218)
Supplement: Supplementary file 1 [file Table_1.DOCX]

Supplementary Material

Quality appraisal of clinical practice guidelines for motor neuron diseases or related disorders using the AGREE II instrument

Jia-Yin Ou^1, 2^, Jun-Jun Liu^3^, Jing Xu^1^, Jia-Yu Li^2^, Yang Liu^4^, You-Zhang Liu^3^, Li-Ming Lu^1*^, Hua-Feng Pan^2*^, Lin Wang^1*^

*** Correspondence: * Correspondence:**

Li-Ming Lu

[lulimingleon@126.com](mailto:lulimingleon@126.com)

Hua-Feng Pan

[gzphf@gzucm.edu.cn](mailto:gzphf@gzucm.edu.cn)

Lin Wang

[wanglin16@gzucm.edu.cn](mailto:wanglin16@gzucm.edu.cn)

**1 PubMed Search Strategy**

| #1 "Guidelines as Topic"[Mesh] |
| --- |
| #2 "Guideline" [Publication Type] |
| #3 guideline*[Title/Abstract] OR consensus[Title/Abstract] OR recommendation*[Title/Abstract] OR guidance[Title/Abstract] OR standard[Title] |
| #4 #1 OR #2 OR #3 |
| #5 "Motor Neuron Disease"[Mesh] |
| #6 motor neuron disease*[Title/Abstract] OR motor neurone disease*[Title/Abstract] OR MND[Title/Abstract] OR amyotrophic lateral sclerosis[Title/Abstract] OR ALS[Title/Abstract] OR progressive bulbar palsy[Title/Abstract] OR primary lateral sclerosis[Title/Abstract] OR primary lateral scleroses*[Title/Abstract] OR progressive muscular atrophy[Title/Abstract] OR motoneuron* disease[Title/Abstract] OR motorneuron* disease[Title/Abstract] OR charcot disease[Title/Abstract] OR Lou Gehrig* disease*[Title/Abstract] OR Lou Gehrig* syndrome*[Title/Abstract] OR Monomelic amyotrophy[Title/Abstract] OR Hirayama Disease[Title/Abstract] OR Progressive pseudobulbar palsy[Title/Abstract] |
| #7 #5 OR #6 |
| #8 "Muscular Atrophy, Spinal"[Mesh] |
| #9 spinal muscular atroph*[Title/Abstract] OR SMA[Title/Abstract] OR muscular disorders, atrophic[Title/Abstract] OR Werdnig Hofmann[Title/Abstract] OR Kugelberg Welander[Title/Abstract] OR Dubowitz disease[Title/Abstract] |
| #10 #8 OR #9 |
| #11 "postpoliomyelitis syndrome"[Mesh] |
| #12 post polio*[Title/Abstract] OR postpolio*[Title/Abstract] OR Post-Polio*[Title/Abstract] OR Postpoliomyelitis[Title/Abstract] OR PPS[Title/Abstract] |
| #13 #11 OR #12 |
| #14 #4 AND #7 |
| #15 #4 AND #10 |
| #16 #4 AND #13 |
| #17 #14 OR #15 OR #16 |
| #18 #17 Filters: from 2006/1/1 - 2022/9/4 |

Results: 1836

**2 Embase Search Strategy**

| #1 'practice guideline'/exp |
| --- |
| #2  guideline:it |
| #3 guideline*:ab,ti OR consensus:ab,ti OR recommendation*:ab,ti OR guidance:ab,ti OR standard:ti |
| #4 #1 OR #2 OR #3 |
| #5 'motor neuron disease'/exp |
| #6  'motor neuron disease*':ab,ti OR 'motor neurone disease*':ab,ti OR mnd:ab,ti OR 'amyotrophic lateral sclerosis':ab,ti OR als:ab,ti OR 'progressive bulbar palsy':ab,ti OR 'primary lateral sclerosis':ab,ti OR 'primary lateral scleroses*':ab,ti OR 'progressive muscular atrophy':ab,ti OR 'motoneuron* disease':ab,ti OR 'motorneuron* disease':ab,ti OR 'charcot disease':ab,ti OR 'lou gehrig* disease*':ab,ti OR 'lou gehrig* syndrome*':ab,ti OR 'monomelic amyotrophy':ab,ti OR 'hirayama disease':ab,ti OR 'progressive pseudobulbar palsy':ab,ti |
| #7 #5 OR #6 |
| #8 'spinal muscular atrophy'/exp |
| #9 'spinal muscular atroph*':ab,ti OR sma:ab,ti OR 'muscular disorders, atrophic':ab,ti OR 'werdnig hofmann':ab,ti OR 'kugelberg welander':ab,ti OR 'dubowitz disease':ab,ti |
| #10 #8 OR #9 |
| #11 'postpoliomyelitis syndrome'/exp |
| #12 postpolio*:ab,ti OR 'post polio*':ab,ti OR postpoliomyelitis:ab,ti OR pps:ab,ti |
| #13 #11 OR #12 |
| #14 #4 AND #7 |
| #15 #4 AND #10 |
| #16 #4 AND #13 |
| #17 #14 OR #15 OR #16 |
| #18 #17 AND [2006-2022]/py |

Result：4659

**3 CINAHL Search Strategy**

| S19 | S18 Limiters - Published Date: 20060101-20220904 |
| --- | --- |
| S18 | S15 OR S16 OR S17 |
| S17 | S3 AND S14 |
| S16 | S3 AND S11 |
| S15 | S3 AND S8 |
| S14 | S12 OR S13 |
| S13 | TI post polio* OR AB post polio* OR TI postpolio* OR AB postpolio* OR TI Post-Polio* OR AB Post-Polio* OR TI Postpoliomyelitis OR AB Postpoliomyelitis OR TI PPS OR AB PPS |
| S12 | (MH "Postpoliomyelitis Syndrome") |
| S11 | S9 OR S10 |
| S10 | TI spinal muscular atroph* OR AB spinal muscular atroph* OR TI SMA OR AB SMA OR TI muscular disorders, atrophic OR AB muscular disorders, atrophic OR TI Werdnig Hofmann OR AB Werdnig Hofmann OR TI Kugelberg Welander OR AB Kugelberg Welander OR TI Dubowitz disease OR AB Dubowitz disease |
| S9 | (MH "Muscular Atrophy, Spinal") |
| S8 | S4 OR S5 OR S6 OR S7 |
| S7 | TI Lou Gehrig* disease* OR AB Lou Gehrig* disease* OR TI Lou Gehrig* syndrome* OR AB Lou Gehrig* syndrome* OR TI Monomelic amyotrophy OR AB Monomelic amyotrophy OR TI Hirayama Disease OR AB Hirayama Disease OR TI Progressive pseudobulbar palsy OR AB Progressive pseudobulbar palsy |
| S6 | TI primary lateral sclerosis OR AB primary lateral sclerosis OR TI primary lateral scleroses* OR AB primary lateral scleroses* OR TI progressive muscular atrophy OR AB progressive muscular atrophy OR TI motoneuron* disease OR AB motoneuron* disease OR TI motorneuron* disease OR AB motorneuron* disease OR TI charcot disease OR AB charcot disease |
| S5 | TI motor neuron disease* OR AB motor neuron disease* OR TI motor neurone disease* OR AB motor neurone disease* OR TI MND OR AB MND OR TI amyotrophic lateral sclerosis OR AB amyotrophic lateral sclerosis OR TI ALS OR AB ALS OR TI progressive bulbar palsy OR AB progressive bulbar palsy |
| S4 | (MH "Motor Neuron Diseases") |
| S3 | S1 OR S2 |
| S2 | TI guideline* OR AB guideline* OR TI consensus OR AB consensus OR TI recommendation* OR AB recommendation* OR TI guidance OR AB guidance OR TI standard |
| S1 | (MH "Practice Guidelines") |

Results: 1055

**4 PEDro Search Strategy**

Abstract & Title: motor neuron disease AND Method: practice guideline AND Published since: 2001

Results: 0

Abstract & Title: amyotrophic lateral sclerosis AND Method: practice guideline AND Published since: 2001

Results: 2

Abstract & Title: progressive bulbar palsy AND Method: practice guideline AND Published since: 2001

Results: 0

Abstract & Title: Progressive muscular atrophy AND Method: practice guideline AND Published since: 2001

Results: 0

Abstract & Title: Primary lateral sclerosis AND Method: practice guideline AND Published since: 2001

Results: 0

Abstract & Title: Spinal Muscular Atrophy AND Method: practice guideline AND Published since: 2001

Results: 0

Abstract & Title: postpolio syndrome AND Method: practice guideline AND Published since: 2001

Results: 0

Abstract & Title: Postpoliomyelitis Syndrome AND Method: practice guideline AND Published since: 2001

Results: 0

**5 NICE Search Strategy**

motor neuron disease (Filters: guidance)

Results: 3

amyotrophic lateral sclerosis (Filters: guidance)

Results: 4

progressive bulbar palsy

Results: 1

Progressive muscular atrophy (Filters: guidance)

Results: 1

Primary lateral sclerosis (Filters: guidance)

Results: 1

Spinal Muscular Atrophy (Filters: guidance)

Results: 5

postpolio syndrome

Results: 1

Postpoliomyelitis Syndrome

Results: 0

**6 NHMRC Search Strategy**

Search Terms: _text_:(motor neuron disease) OR _text_:(amyotrophic lateral sclerosis) OR _text_:(progressive bulbar palsy) OR _text_:(Progressive muscular atrophy) OR _text_:(Primary lateral sclerosis) OR _text_:(Spinal Muscular Atrophy) OR _text_:(postpolio syndrome) OR _text_:(Postpoliomyelitis Syndrome)

Subjects ANZSRC-FOR: [Neurosciences](https://researchdata.edu.au/search/)

Time Period (from): [2001](https://researchdata.edu.au/search/)

Results: 25

**7 AHRQ Search Strategy**

motor neuron (Filters: title)

Results: 4

amyotrophic lateral sclerosis (Filters: title)

Results: 9

progressive bulbar palsy (Filters: title)

Results: 10

Progressive muscular atrophy (Filters: title)

Results: 0

lateral sclerosis (Filters: title)

Results: 9

Spinal Muscular Atrophy (Filters: title)

Results: 16

postpolio (Filters: title)

Results: 0

Postpoliomyelitis (Filters: title)

Results: 0

**8 GIN Search Strategy**

motor neuron disease

Results: 1

amyotrophic lateral sclerosis

Results: 3

progressive bulbar palsy

Results: 0

Progressive muscular atrophy

Results: 0

Primary lateral sclerosis

Results: 0

Spinal Muscular Atrophy

Results: 0

postpolio syndrome

Results: 0

Postpoliomyelitis Syndrome

Results: 0

**9 SIGN Search Strategy**

motor neuron

Results: 0

amyotrophic lateral sclerosis

Results: 0

bulbar palsy

Results: 0

muscular atrophy

Results: 0

lateral sclerosis

Results: 0

Spinal Muscular Atrophy

Results: 0

postpolio

Results: 0

Postpoliomyelitis

Results: 0

**10 WHO Search Strategy**

motor neuron disease

Results: 0

amyotrophic lateral sclerosis

Results: 0

progressive bulbar palsy

Results: 0

Progressive muscular atrophy

Results: 0

Primary lateral sclerosis

Results: 0

Spinal Muscular Atrophy

Results: 0

postpolio syndrome

Results: 0

Postpoliomyelitis Syndrome

Results: 0
