# Supplementary material for: Quality appraisal of clinical practice guidelines for motor neuron diseases or related disorders using the AGREE II instrument
Source: Front Neurol. 2023 Jul 17;14:1180218. doi: 10.3389/fneur.2023.1180218 (PMC10388716; doi:10.3389/fneur.2023.1180218)
Supplement: Supplementary file 2 [file Table_2.DOC]

Supplementary Table 1. Supplementary characteristics of included CPGs.

| CPG ID (development organization*, year) | title | Journal (if available) | Used grading system | Funding sources |
| --- | --- | --- | --- | --- |
| Pitarch et al.，2022 (1) | Delphi consensus on recommendations for the  treatment of spinal muscular atrophy in Spain  (RET-AME consensus) | Neurologia (Engl Ed) | No | Not stated |
| Neuromuscular Disorders Task Force，2022 (2) | Proposals from a French expert panel for respiratory care in ALS  patients | Respir Med Res | No | No |
| Shoesmith et al., 2020 (3) | Canadian best practice recommendations for the management of amyotrophic lateral sclerosis | CMAJ | Yes | ALS Society of  Canada , Canadian ALS Research Network |
| FILSLAN，2020 (4) | Pre-symptomatic  diagnosis  in  ALS | Rev Neurol (Paris) | No | Not stated |
| CTS, 2019 (5) | Home mechanical ventilation for patients with Amyotrophic Lateral Sclerosis: A Canadian Thoracic Society clinical practice guideline | Can Respir J | Yes | No |
| NEALS bulbar subcommittee, 2019(6) | Provisional best practices guidelines for the evaluation of bulbar dysfunction in amyotrophic lateral sclerosis | Muscle Nerve | No | CNS，NEALS consortium |
| BMA，2018 (7) | Spinal muscular atrophy 5Q - Treatment with nusinersen | Rev Assoc Med Bras (1992) | Yes | Not stated |
| SMA Care Group，2018 (8, 9) | Diagnosis and management of spinal muscular atrophy: Part 1: Recommendations for diagnosis, rehabilitation, orthopedic and nutritional care  Diagnosis and management of spinal muscular atrophy: Part 2: Pulmonary and acute care; medications, supplements and immunizations; other organ systems; and ethics | Neuromuscul Disord | Yes | ENMC, TREAT-NMD, SMA Europe, SMA support UK, SMA Foundation, Cure SMA, Italian Telethon |
| NICE，2019 (10) | Motor neurone disease: assessment and management | NA | Yes | Motor Neurone Disease Association,Brain and Spine Foundation, Marie Curie |
| CORNEMUS  , 2012 (11) | Recommendations for the diagnosis and management of typical childhood spinal muscular atrophy | Rev Neurol (Paris) | No | Not stated |
| EFNS, 2012 (12) | EFNS guidelines on the Clinical Management of Amyotrophic Lateral Sclerosis (MALS) – revised report of an EFNS task force | Eur J Neurol | Yes | No |
| EFNS, 2010 (13) | EFNS guidelines on the use of neuroimaging in the management of motor neuron diseases | Eur J Neurol | Yes | Not stated |
| AAN，2009 (14, 15) | Practice parameter update: the care of the patient with amyotrophic lateral sclerosis: multidisciplinary care, symptom management, and cognitive/behavioral impairment (an evidence-based review): report of the Quality Standards Subcommittee of the American Academy of Neurology  Practice parameter update: the care of the patient with amyotrophic lateral sclerosis: drug, nutritional, and respiratory therapies (an evidence-based review): report of the Quality Standards Subcommittee of the American Academy of Neurology | Neurology | Yes | No |
| ACOG，2009 (16) | ACOG committee opinion No. 432: spinal muscular atrophy | Obstet Gynecol | No | Not stated |
| IFCN, 2008 (17) | Electrodiagnostic criteria for diagnosis of ALS | Clin Neurophysiol | No | IFCN, Japanese Ministry of Education, Science, Culture and Sports (Centre of Excellence or COE grant) and Ministry of Health, Welfare and Labour,Eisai Pharmaceutical Co., Nihon Pharmaceutical Co., Takeda Pharmaceutical Co., Mitsubishi WelPharma Co.,Astelas Pharma Co. |

*First author given where there is no stated organisation; CPG: clinical practice guideline; FILSLAN: Filie` re Nationale de Sante´ SLA et autres Maladies rares du Neurone Moteur （National Heathcare Network for ALS and other rare motor neuron diseases）; CTS: Canadian Thoracic society; NEALS: Northeast ALS; USA：United States of America； SMA: spinal muscular atrophy; NICE: National Institute for Health and Clinical Excellence; CORNEMUS: network of French centers of expertise for neuromuscular diseases; EFNS: European Federation of Neurological Societies; AAN: American Academy of Neurology; ACOG: The American College of Obstetricians and Gynecologists; IFCN: International Federation of Clinical Neurophysiolog; ALS: [amyotrophic lateral sclerosis](https://www.ncbi.nlm.nih.gov/mesh/68000690); CNS: Cytokinetics and the Center for Neurological Studies; ENMC: European Neuromuscular Consortium; UK: United Kingdom; BMA: Brazilian Medical Association.

Supplementary Table 2. [Descriptive statistic](javascript:;)s of characteristics of included CPGs.

| Characteristic | n (%) |
| --- | --- |
| Disease |  |
| MND | 10 (66.7) |
| SMA | 5 (33.3) |
| Year |  |
| ≤ 2015 | 6 (40.0) |
| > 2015 | 9 (60.0) |
| Type of development organization |  |
| Expert panel | 5 (33.3) |
| Government | 2 (13.3) |
| Medical society | 8 (53.3) |
| Country/region of origin |  |
| European country | 5 (33.3) |
| North American country | 5 (33.3) |
| Europe | 2 (13.3) |
| International | 2 (13.3) |
| Brazil | 1 (6.7) |
| Version |  |
| First | 9 (60.0) |
| Updated | 6 (40.0) |
| Age range of target population |  |
| Adult | 3 (20.0) |
| Children | 1 (6.7) |
| All ages | 5 (33.3) |
| Not stated | 6 (40.0) |
| Development method |  |
| CB | 7 (46.7) |
| EB | 8 (53.3) |
| Stated search dates |  |
| Yes | 7 (46.7) |
| No | 8 (53.3) |
| Used CPG quality tool |  |
| Yes | 3 (20.0) |
| No | 12 (80.0) |
| Included CPG methodologist |  |
| Yes | 1 (6.7) |
| No | 7 (46.7) |
| Not stated | 7 (46.7) |
| Used grading system |  |
| Yes | 8 (53.3) |
| No | 7 (46.7) |
| With funding sources |  |
| Yes | 5 (33.3) |
| No | 4 (26.7) |
| Not stated | 6 (40.0) |

CPG: clinical practice guideline; MND: motor neuron disease; SMA: spinal muscular atrophy; EB: evidence-based; CB: consensus-based.

Supplementary Table 3. Characteristics of CPGs between MND and SMA.

| Characteristic | MND, n (%) | SMA, n (%) | P-value |
| --- | --- | --- | --- |
| Year |  |  | 1.000 |
| ≤ 2015 | 4 (40.0) | 2 (40.0) |  |
| > 2015 | 6 (60.0) | 3 (60.0) |  |
| Type of development organization |  |  | 0.324 |
| Expert panel | 2 (20.0) | 3 (60.0) |  |
| Government | 2 (20.0) | 0 (0.0) |  |
| Medical society | 6 (60.0) | 2 (40.0) |  |
| Country/region of origin |  |  | - |
| European country | 3 (30.0) | 2 (40.0) |  |
| North American country | 4 (40.0) | 1 (20.0) |  |
| Europe | 2 (20.0) | 0 (0.0) |  |
| International | 1 (10.0) | 1 (20.0) |  |
| Brazil | 0 (0.0) | 1 (20.0) |  |
| Version |  |  | 0.580 |
| First | 5 (50.0) | 4 (80.0) |  |
| Updated | 5 (50.0) | 1 (20.0) |  |
| Age range of target population |  |  | - |
| Adult | 3 (30.0) | 0 (0.0) |  |
| Children | 0 (0.0) | 1 (20.0) |  |
| All ages | 1 (10.0) | 4 (80.0) |  |
| Not stated | 6 (60.0) | 0 (0.0) |  |
| Development method |  |  | 0.608 |
| CB | 4 (40.0) | 3 (60.0) |  |
| EB | 6 (60.0) | 2 (40.0) |  |
| Stated search dates |  |  | 0.026 |
| Yes | 7 (70.0) | 0 (0.0) |  |
| No | 3 (30.0) | 5 (100.0) |  |
| Used CPG quality tool |  |  | 0.505 |
| Yes | 3 (30.0) | 0 (0.0) |  |
| No | 7 (70.0) | 5 (100.0) |  |
| Included CPG methodologist |  |  | 1.000 |
| Yes | 1 (10.0) | 0 (0.0) |  |
| No | 4 (40.0) | 3 (60.0) |  |
| Not stated | 5 (50.0) | 2 (40.0) |  |
| Used grading system |  |  | 0.608 |
| Yes | 6 (60.0) | 2 (40.0) |  |
| No | 4 (40.0) | 3 (60.0) |  |
| With funding sources |  |  | 0.121 |
| Yes | 4 (40.0) | 1 (20.0) |  |
| No | 4 (40.0) | 0 (0.0) |  |
| Not stated | 2 (20.0) | 4 (80.0) |  |

CPG: clinical practice guideline; MND: motor neuron disease; SMA: spinal muscular atrophy; EB: evidence-based; CB: consensus-based.

Supplementary Table 4. AGREE II domain and overall assessment score of CPGs between MND and SMA.

| AGREE II domain and overall assessment | MND, %, Mean(SD) Median (Q1-Q3) / N(%) | SMA, %, Mean(SD) Median (Q1-Q3) / N(%) | P-value |
| --- | --- | --- | --- |
| n | 10 | 5 |  |
| Domain 1 | 73.9 (20.1) 77.5 (57.2-91.2) | 56.6 (28.6) 61.0 (37.0-67.0) | 0.194 |
| Domain 2 | 43.3 (24.7) 40.0 (27.8-58.8) | 34.2 (16.5) 28.0 (26.0-46.0) | 0.540 |
| Domain 3 | 46.7 (30.9) 46.0 (26.2-69.2) | 26.2 (23.4) 15.0 (13.0-28.0) | 0.244 |
| Domain 4 | 84.6 (12.7) 90.0 (71.8-95.2) | 71.6 (16.1) 67.0 (63.0-85.0) | 0.110 |
| Domain 5 | 29.5 (28.6) 22.5 (9.8-33.2) | 14.2 (4.8) 13.0 (11.0-15.0) | 0.358 |
| Domain 6 | 68.5 (33.4) 80.5 (47.8-96.2) | 30.6 (21.1) 42.0 (17.0-47.0) | 0.036 |
| Overall rating | 51.2 (27.4) 53.0 (30.8-73.8) | 31.0 (10.0) 28.0 (22.0-39.0) | 0.177 |
| Quality |  |  | 0.505 |
| Low | 7 (70.0) | 5 (100.0) |  |
| High | 3 (30.0) | 0 (0.0) |  |

CPG: clinical practice guideline; AGREE: Appraisal of Guidelines for Research and Evaluation; MND: motor neuron disease; SMA: spinal muscular atrophy.

Supplementary Table 5. Overall assessment of included CPGs using other criteria.

| CPG ID | Overall rating (%) | Quality (high/low) |
| --- | --- | --- |
| Pitarch et al.，2022 (1) | 43% | low |
| Neuromuscular Disorders Task Force，2022 (2) | 52% | low |
| Shoesmith et al., 2020 (3) | 84% | low |
| FILSLAN，2020 (4) | 30% | low |
| CTS, 2019 (5) | 87% | high |
| NEALS bulbar subcommittee, 2019(6) | 40% | low |
| BMA，2018 (7) | 51% | low |
| SMA Care Group，2018 (8, 9) | 40% | low |
| NICE，2019 (10) | 81% | high |
| CORNEMUS  , 2012 (11) | 33% | low |
| EFNS, 2012 (12) | 62% | low |
| EFNS, 2010 (13) | 52% | low |
| AAN，2009 (14, 15) | 60% | low |
| ACOG，2009 (16) | 27% | low |
| IFCN, 2008 (17) | 31% | low |

AGREE: Appraisal of Guidelines for Research and Evaluation; CPG: clinical practice guideline; FILSLAN: Filie` re Nationale de Sante´ SLA et autres Maladies rares du Neurone Moteur (National Heathcare Network for ALS and other rare motor neuron diseases）; CTS: Canadian Thoracic society; NEALS: Northeast ALS; SMA: spinal muscular atrophy; NICE: National Institute for Health and Clinical Excellence; CORNEMUS: network of French centers of expertise for neuromuscular diseases; EFNS: European Federation of Neurological Societies; AAN: American Academy of Neurology；ACOG: The American College of Obstetricians and Gynecologists; IFCN: International Federation of Clinical Neurophysiolog; BMA: Brazilian Medical Association.

Supplementary Table 6. Relationship between characteristics and AGREE II domain score and overall assessment of included CPGs published after 2015.

| Characteristic | Statistics | Domain 1, % (β, 95%CI, P-value) | Domain 2, % (β, 95%CI, P-value) | Domain 3, % (β, 95%CI, P-value) | Domain 4, % (β, 95%CI, P-value) | Domain 5, % (β, 95%CI, P-value) | Domain 6, % (β, 95%CI, P-value) | Overall rating, % (β, 95%CI, P-value) | Quality (OR, 95%CI, P-value) |
| --- | --- | --- | --- | --- | --- | --- | --- | --- | --- |
| Disease |  |  |  |  |  |  |  |  |  |
| MND | 6 (66.7%) | Refrence | Refrence | Refrence | Refrence | Refrence | Refrence | Refrence | Refrence |
| SMA | 3 (33.3%) | -12.2 (-46.6, 22.3) 0.511 | -5.7 (-43.0, 31.7) 0.775 | -15.8 (-64.5, 32.9) 0.544 | -8.8 (-27.6, 9.9) 0.386 | -25.2 (-62.8, 12.4) 0.231 | -36.3 (-75.9, 3.3) 0.115 | -17.7 (-56.6, 21.3) 0.403 | ––* |
| Type of development organization |  |  |  |  |  |  |  |  |  |
| Expert panel | 4 (44.4%) | Refrence | Refrence | Refrence | Refrence | Refrence | Refrence | Refrence | Refrence |
| Government | 2 (22.2%) | 9.5 (-30.4, 49.4) 0.657 | -16.5 (-62.9, 29.9) 0.512 | -0.5 (-63.5, 62.5) 0.988 | 4.5 (-20.9, 29.9) 0.740 | 14.0 (-39.8, 67.8) 0.628 | -7.7 (-70.3, 54.8) 0.816 | -2.8 (-56.6, 51.1) 0.923 | 3.0 (0.1, 107.5) 0.547 |
| Medical society | 3 (33.3%) | 27.5 (-7.7, 62.7) 0.176 | -17.7 (-58.6, 23.3) 0.430 | 21.0 (-34.5, 76.5) 0.486 | -4.3 (-26.7, 18.0) 0.717 | 13.0 (-34.4, 60.4) 0.610 | -10.4 (-65.6, 44.8) 0.724 | 6.4 (-41.0, 53.9) 0.800 | 1.5 (0.1, 40.6) 0.810 |
| Country/region of origin |  |  |  |  |  |  |  |  |  |
| European country | 4 (44.4%) | Refrence | Refrence | Refrence | Refrence | Refrence | Refrence | Refrence | Refrence |
| North American country | 3 (33.3%) | 30.6 (-2.3, 63.5) 0.118 | 17.3 (-23.0, 57.7) 0.432 | 39.3 (-9.9, 88.6) 0.169 | 4.4 (-14.9, 23.7) 0.669 | 14.3 (-29.1, 57.8) 0.542 | 8.0 (-40.5, 56.5) 0.757 | 27.7 (-14.2, 69.6) 0.243 | 6.0 (0.2, 162.5) 0.287 |
| Other | 2 (22.2%) | 21.7 (-15.5, 59.0) 0.296 | -6.0 (-51.7, 39.7) 0.806 | 17.0 (-38.9, 72.9) 0.573 | -14.2 (-36.1, 7.6) 0.249 | -19.5 (-68.7, 29.7) 0.467 | -32.0 (-87.0, 23.0) 0.297 | 2.5 (-45.0, 50.0) 0.921 | ––* |
| Version |  |  |  |  |  |  |  |  |  |
| First | 5 (55.6%) | Refrence | Refrence | Refrence | Refrence | Refrence | Refrence | Refrence | Refrence |
| Updated | 4 (44.4%) | 9.0 (-24.1, 42.0) 0.612 | 11.8 (-22.8, 46.4) 0.525 | 14.6 (-31.7, 60.9) 0.556 | 4.8 (-13.6, 23.3) 0.623 | 32.5 (0.8, 64.2) 0.085 | 17.9 (-25.6, 61.4) 0.446 | 22.4 (-12.8, 57.7) 0.252 | 4.0 (0.2, 75.7) 0.355 |
| Age range of target population |  |  |  |  |  |  |  |  |  |
| Adult | 2 (22.2%) | Refrence | Refrence | Refrence | Refrence | Refrence | Refrence | Refrence | Refrence |
| Children | 1 (11.1%) | -2.0 (-53.3, 49.3) 0.942 | -40.5 (-79.8, -1.2) 0.100 | -17.0 (-82.9, 48.9) 0.635 | -30.5 (-51.1, -9.9) 0.034 | -69.5 (-94.9, -44.1) 0.003 | -33.5 (-124.4, 57.4) 0.502 | -39.5 (-89.3, 10.3) 0.181 | ––* |
| All ages | 3 (33.3%) | -32.0 (-70.2, 6.2) 0.162 | -2.8 (-32.1, 26.5) 0.857 | -38.3 (-87.5, 10.8) 0.187 | -11.8 (-27.2, 3.6) 0.192 | -55.5 (-74.4, -36.6) 0.002 | -27.5 (-95.2, 40.2) 0.462 | -33.5 (-70.6, 3.6) 0.137 | ––* |
| Not stated | 3 (33.3%) | -37.0 (-75.2, 1.2) 0.116 | -43.8 (-73.1, -14.5) 0.033 | -64.3 (-113.5, -15.2) 0.050 | -25.8 (-41.2, -10.4) 0.022 | -62.5 (-81.4, -43.6) 0.001 | -24.2 (-91.9, 43.6) 0.515 | -57.5 (-94.6, -20.4) 0.029 | ––* |
| Development method |  |  |  |  |  |  |  |  |  |
| CB | 4 (44.4%) | Refrence | Refrence | Refrence | Refrence | Refrence | Refrence | Refrence | Refrence |
| EB | 5 (55.6%) | 35.6 (14.5, 56.7) 0.013 | 27.8 (-1.3, 56.9) 0.103 | 52.4 (25.1, 79.8) 0.007 | 10.0 (-7.3, 27.3) 0.295 | 25.5 (-9.5, 60.6) 0.196 | 12.2 (-32.3, 56.8) 0.606 | 40.1 (14.9, 65.3) 0.017 | ––* |
| Stated search dates |  |  |  |  |  |  |  |  |  |
| Yes | 3 (33.3%) | Refrence | Refrence | Refrence | Refrence | Refrence | Refrence | Refrence | Refrence |
| No | 6 (66.7%) | -10.8 (-45.5, 23.8) 0.560 | -22.8 (-56.4, 10.7) 0.224 | -33.2 (-76.8, 10.5) 0.180 | -9.2 (-27.8, 9.5) 0.367 | -25.3 (-62.9, 12.2) 0.228 | -57.7 (-79.3, -36.0) 0.001 | -32.3 (-65.7, 1.0) 0.099 | 0.1 (0.0, 2.5) 0.161 |
| Used CPG quality tool |  |  |  |  |  |  |  |  |  |
| Yes | 3 (33.3%) | Refrence | Refrence | Refrence | Refrence | Refrence | Refrence | Refrence | Refrence |
| No | 6 (66.7%) | -32.8 (-58.8, -6.9) 0.042 | -41.3 (-63.1, -19.5) 0.008 | -58.2 (-83.7, -32.6) 0.003 | -23.7 (-32.9, -14.4) 0.002 | -48.3 (-70.2, -26.4) 0.003 | -41.2 (-78.1, -4.2) 0.065 | -51.8 (-66.4, -37.3) <0.001 | ––* |
| Included CPG methodologist |  |  |  |  |  |  |  |  |  |
| Yes | 1 (11.1%) | Refrence | Refrence | Refrence | Refrence | Refrence | Refrence | Refrence | Refrence |
| No | 6 (66.7%) | -28.2 (-82.1, 25.8) 0.346 | -17.8 (-62.9, 27.3) 0.468 | -29.8 (-107.1, 47.4) 0.478 | -17.7 (-42.5, 7.1) 0.212 | -40.0 (-90.3, 10.3) 0.170 | -4.5 (-81.5, 72.5) 0.913 | -27.0 (-84.1, 30.1) 0.389 | ––* |
| Not stated | 2 (22.2%) | -29.0 (-90.2, 32.2) 0.389 | -52.5 (-103.6, -1.4) 0.091 | -41.5 (-129.1, 46.1) 0.389 | -30.0 (-58.1, -1.9) 0.081 | -67.0 (-124.0, -10.0) 0.061 | -20.0 (-107.3, 67.3) 0.669 | -50.5 (-115.2, 14.2) 0.177 | ––* |
| Used grading system |  |  |  |  |  |  |  |  |  |
| Yes | 5 (55.6%) | Refrence | Refrence | Refrence | Refrence | Refrence | Refrence | Refrence | Refrence |
| No | 4 (44.4%) | -35.6 (-56.7, -14.5) 0.013 | -27.8 (-56.9, 1.3) 0.103 | -52.5 (-79.8, -25.1) 0.007 | -10.0 (-27.3, 7.3) 0.295 | -25.6 (-60.6, 9.5) 0.196 | -12.3 (-56.8, 32.3) 0.606 | -40.1 (-65.3, -14.9) 0.017 | ––* |
| With funding sources |  |  |  |  |  |  |  |  |  |
| Yes | 4 (44.4%) | Refrence | Refrence | Refrence | Refrence | Refrence | Refrence | Refrence | Refrence |
| No | 2 (22.2%) | -9.5 (-50.3, 31.3) 0.664 | -9.7 (-52.8, 33.3) 0.673 | 2.5 (-57.7, 62.7) 0.938 | -1.7 (-26.8, 23.3) 0.896 | 20.8 (-22.9, 64.4) 0.387 | 45.5 (-1.9, 92.9) 0.109 | 7.0 (-37.2, 51.2) 0.767 | 1.0 (0.0, 29.8) 1.000 |
| Not stated | 3 (33.3%) | -25.8 (-61.8, 10.1) 0.209 | -27.4 (-65.4, 10.5) 0.207 | -27.5 (-80.5, 25.5) 0.349 | -8.4 (-30.5, 13.7) 0.484 | -23.8 (-62.2, 14.7) 0.272 | -6.2 (-48.0, 35.7) 0.782 | -29.3 (-68.4, 9.7) 0.191 | ––* |

*The model failed because of the small sample size; AGREE: Appraisal of Guidelines for Research and Evaluation; CPG: clinical practice guideline; CI: confidence interval; OR: odds ratio; MND: motor neuron disease; SMA: spinal muscular atrophy; EB: evidence-based; CB: consensus-based.

Supplementary Table 7. Relationship between characteristics and AGREE II domain score and overall assessment of included evidence-based CPGs.

| Characteristic | Statistics | Domain 1, % (β, 95%CI, P-value) | Domain 2, % (β, 95%CI, P-value) | Domain 3, % (β, 95%CI, P-value) | Domain 4, % (β, 95%CI, P-value) | Domain 5, % (β, 95%CI, P-value) | Domain 6, % (β, 95%CI, P-value) | Overall rating, % (β, 95%CI, P-value) | Quality (OR, 95%CI, P-value) |
| --- | --- | --- | --- | --- | --- | --- | --- | --- | --- |
| Disease |  |  |  |  |  |  |  |  |  |
| MND | 6 (75.0%) | Refrence | Refrence | Refrence | Refrence | Refrence | Refrence | Refrence | Refrence |
| SMA | 2 (25.0%) | -3.2 (-28.9, 22.5) 0.817 | -22.0 (-52.2, 8.2) 0.204 | -19.3 (-55.8, 17.1) 0.339 | -28.8 (-34.2, -23.5) <0.001 | -25.7 (-76.0, 24.7) 0.356 | -51.7 (-84.5, -18.9) 0.021 | -28.0 (-51.4, -4.6) 0.057 | ––* |
| Year |  |  |  |  |  |  |  |  |  |
| ≤ 2015 | 3 (37.5%) | Refrence | Refrence | Refrence | Refrence | Refrence | Refrence | Refrence | Refrence |
| > 2015 | 5 (62.5%) | 17.9 (-0.2, 36.0) 0.100 | 16.8 (-11.4, 45.0) 0.288 | 23.2 (-7.0, 53.4) 0.182 | -12.3 (-31.1, 6.4) 0.245 | 32.1 (-9.2, 73.4) 0.178 | -15.3 (-60.9, 30.2) 0.534 | 10.9 (-16.7, 38.6) 0.467 | ––* |
| Type of development organization |  |  |  |  |  |  |  |  |  |
| Expert panel | 2 (25.0%) | Refrence | Refrence | Refrence | Refrence | Refrence | Refrence | Refrence | Refrence |
| Government | 1 (12.5%) | 19.5 (-18.6, 57.6) 0.362 | 2.5 (-41.2, 46.2) 0.915 | 16.5 (-46.4, 79.4) 0.629 | 16.0 (-20.2, 52.2) 0.426 | 49.0 (-26.0, 124.0) 0.257 | 8.5 (-75.0, 92.0) 0.850 | 17.0 (-33.1, 67.1) 0.536 | ––* |
| Medical society | 5 (62.5%) | 1.5 (-24.6, 27.6) 0.915 | -24.5 (-54.4, 5.4) 0.169 | -0.1 (-43.1, 42.9) 0.997 | 7.4 (-17.3, 32.1) 0.583 | 1.0 (-50.3, 52.3) 0.971 | 17.9 (-39.1, 74.9) 0.565 | -1.0 (-35.3, 33.3) 0.957 | 0.2 (0.0, 8.6) 0.442 |
| Country/region of origin |  |  |  |  |  |  |  |  |  |
| European country | 1 (12.5%) | Refrence | Refrence | Refrence | Refrence | Refrence | Refrence | Refrence | Refrence |
| North American country | 3 (37.5%) | -12.0 (-44.8, 20.8) 0.505 | -7.0 (-47.8, 33.8) 0.751 | 0.7 (-43.1, 44.5) 0.977 | -2.7 (-32.6, 27.2) 0.868 | -31.3 (-90.2, 27.5) 0.344 | 30.0 (-25.8, 85.8) 0.340 | -0.3 (-25.1, 24.4) 0.980 | ––* |
| Other | 4 (50.0%) | -23.2 (-55.0, 8.5) 0.211 | -29.8 (-69.3, 9.8) 0.200 | -29.5 (-71.9, 12.9) 0.231 | -16.8 (-45.7, 12.2) 0.308 | -61.0 (-118.0, -4.0) 0.090 | -15.0 (-69.0, 39.0) 0.610 | -30.7 (-54.7, -6.8) 0.053 | ––* |
| Version |  |  |  |  |  |  |  |  |  |
| First | 3 (37.5%) | Refrence | Refrence | Refrence | Refrence | Refrence | Refrence | Refrence | Refrence |
| Updated | 5 (62.5%) | -11.4 (-32.6, 9.8) 0.333 | -0.3 (-31.5, 31.0) 0.987 | -10.4 (-44.8, 24.0) 0.575 | 7.9 (-12.3, 28.1) 0.471 | 26.3 (-17.6, 70.1) 0.285 | 8.1 (-38.6, 54.9) 0.745 | 5.6 (-23.0, 34.2) 0.715 | 1.3 (0.1, 26.6) 0.851 |
| Age range of target population |  |  |  |  |  |  |  |  |  |
| Adult | 3 (37.5%) | Refrence | Refrence | Refrence | Refrence | Refrence | Refrence | Refrence | Refrence |
| Children | 1 (12.5%) | 3.0 (-24.9, 30.9) 0.843 | -32.7 (-74.4, 9.0) 0.199 | -5.0 (-66.5, 56.5) 0.881 | -29.0 (-50.6, -7.4) 0.058 | -43.3 (-119.5, 32.8) 0.327 | -23.3 (-98.7, 52.0) 0.577 | -28.3 (-76.2, 19.5) 0.310 | ––* |
| All ages | 2 (25.0%) | -12.5 (-34.5, 9.5) 0.329 | 8.8 (-24.1, 41.8) 0.627 | -11.5 (-60.1, 37.1) 0.667 | -12.0 (-29.1, 5.1) 0.241 | -28.3 (-88.5, 31.9) 0.409 | -11.8 (-71.4, 47.7) 0.717 | -11.3 (-49.2, 26.5) 0.589 | 0.5 (0.0, 19.6) 0.711 |
| Not stated | 2 (25.0%) | -25.5 (-47.5, -3.5) 0.086 | -17.2 (-50.1, 15.8) 0.365 | -24.0 (-72.6, 24.6) 0.388 | 5.0 (-12.1, 22.1) 0.598 | -36.3 (-96.5, 23.9) 0.302 | 25.2 (-34.4, 84.7) 0.454 | -13.8 (-51.7, 24.0) 0.513 | ––* |
| Stated search dates |  |  |  |  |  |  |  |  |  |
| Yes | 5 (62.5%) | Refrence | Refrence | Refrence | Refrence | Refrence | Refrence | Refrence | Refrence |
| No | 3 (37.5%) | 6.1 (-16.5, 28.7) 0.617 | -8.3 (-38.8, 22.3) 0.615 | -7.7 (-42.6, 27.1) 0.679 | -18.1 (-33.5, -2.6) 0.062 | 2.0 (-46.6, 50.6) 0.938 | -43.3 (-75.4, -11.3) 0.038 | -14.1 (-40.8, 12.5) 0.339 | 0.8 (0.0, 15.0) 0.851 |
| Used CPG quality tool |  |  |  |  |  |  |  |  |  |
| Yes | 3 (37.5%) | Refrence | Refrence | Refrence | Refrence | Refrence | Refrence | Refrence | Refrence |
| No | 5 (62.5%) | -20.5 (-36.8, -4.2) 0.049 | -34.4 (-49.3, -19.5) 0.004 | -38.7 (-55.9, -21.4) 0.005 | -10.7 (-30.1, 8.6) 0.319 | -52.7 (-76.9, -28.4) 0.005 | -26.0 (-68.4, 16.4) 0.274 | -33.3 (-44.6, -22.0) 0.001 | ––* |
| Included CPG methodologist |  |  |  |  |  |  |  |  |  |
| Yes | 1 (12.5%) | Refrence | Refrence | Refrence | Refrence | Refrence | Refrence | Refrence | Refrence |
| No | 3 (37.5%) | -14.3 (-49.1, 20.4) 0.455 | -4.0 (-38.7, 30.7) 0.830 | -6.3 (-60.7, 48.0) 0.828 | -12.3 (-47.4, 22.7) 0.521 | -29.0 (-83.8, 25.8) 0.347 | 3.3 (-78.3, 85.0) 0.939 | -7.7 (-48.8, 33.5) 0.730 | ––* |
| Not stated | 4 (50.0%) | -21.5 (-55.1, 12.1) 0.266 | -32.0 (-65.5, 1.5) 0.120 | -24.2 (-76.9, 28.4) 0.408 | -9.5 (-43.4, 24.4) 0.607 | -62.7 (-115.8, -9.7) 0.068 | 5.0 (-74.0, 84.0) 0.906 | -25.2 (-65.1, 14.6) 0.269 | ––* |
| With funding sources |  |  |  |  |  |  |  |  |  |
| Yes | 3 (37.5%) | Refrence | Refrence | Refrence | Refrence | Refrence | Refrence | Refrence | Refrence |
| No | 3 (37.5%) | -10.0 (-36.1, 16.1) 0.486 | -19.7 (-46.7, 7.3) 0.213 | -3.7 (-46.5, 39.2) 0.873 | 9.7 (-11.5, 30.8) 0.411 | -1.7 (-52.7, 49.4) 0.951 | 33.7 (-8.7, 76.0) 0.180 | 2.0 (-28.5, 32.5) 0.903 | 0.2 (0.0, 7.5) 0.423 |
| Not stated | 2 (25.0%) | 2.5 (-26.7, 31.7) 0.873 | -33.8 (-64.0, -3.7) 0.079 | -8.5 (-56.4, 39.4) 0.742 | -9.3 (-33.0, 14.3) 0.474 | -35.8 (-92.9, 21.2) 0.273 | -12.8 (-60.2, 34.5) 0.618 | -19.7 (-53.7, 14.4) 0.309 | ––* |

*The model failed because of the small sample size; AGREE: Appraisal of Guidelines for Research and Evaluation; CPG: clinical practice guideline; CI: confidence interval; OR: odds ratio; MND: motor neuron disease; SMA: spinal muscular atrophy.

Supplementary Table 8. Relationship between characteristics and AGREE II domain score and overall assessment of included MND CPGs.

| Characteristic | Statistics | Domain 1, % (β, 95%CI, P-value) | Domain 2, % (β, 95%CI, P-value) | Domain 3, % (β, 95%CI, P-value) | Domain 4, % (β, 95%CI, P-value) | Domain 5, % (β, 95%CI, P-value) | Domain 6, % (β, 95%CI, P-value) | Overall rating, % (β, 95%CI, P-value) | Quality (OR, 95%CI, P-value) |
| --- | --- | --- | --- | --- | --- | --- | --- | --- | --- |
| Year |  |  |  |  |  |  |  |  |  |
| ≤ 2015 | 4 (40.0%) | Refrence | Refrence | Refrence | Refrence | Refrence | Refrence | Refrence | Refrence |
| > 2015 | 6 (60.0%) | 12.3 (-13.3, 37.9) 0.373 | 12.6 (-19.5, 44.6) 0.464 | 13.7 (-26.6, 54.0) 0.525 | -10.3 (-25.8, 5.3) 0.231 | 28.3 (-4.6, 61.3) 0.130 | 7.9 (-36.6, 52.4) 0.736 | 8.7 (-27.6, 44.9) 0.652 | ––* |
| Type of development organization |  |  |  |  |  |  |  |  |  |
| Expert panel | 2 (20.0%) | Refrence | Refrence | Refrence | Refrence | Refrence | Refrence | Refrence | Refrence |
| Government | 2 (20.0%) | -2.0 (-46.7, 42.7) 0.933 | -21.5 (-72.4, 29.4) 0.435 | -19.0 (-86.0, 48.0) 0.595 | 2.5 (-25.0, 30.0) 0.863 | 7.5 (-54.8, 69.8) 0.820 | -43.0 (-107.2, 21.2) 0.231 | -16.5 (-76.1, 43.1) 0.604 | 1.0 (0.0, 50.4) 1.000 |
| Medical society | 6 (60.0%) | -1.2 (-37.7, 35.3) 0.952 | -22.3 (-63.9, 19.2) 0.327 | -13.3 (-68.0, 41.3) 0.647 | 6.8 (-15.6, 29.2) 0.569 | -5.8 (-56.7, 45.0) 0.829 | -38.2 (-90.6, 14.3) 0.197 | -10.8 (-59.5, 37.8) 0.676 | 0.2 (0.0, 6.7) 0.368 |
| Country/region of origin |  |  |  |  |  |  |  |  |  |
| European country | 3 (30.0%) | Refrence | Refrence | Refrence | Refrence | Refrence | Refrence | Refrence | Refrence |
| North American country | 4 (40.0%) | 18.9 (-10.0, 47.8) 0.240 | 16.4 (-22.7, 55.5) 0.438 | 29.2 (-16.4, 74.9) 0.250 | 9.4 (-10.3, 29.2) 0.381 | 1.4 (-43.6, 46.4) 0.952 | 6.9 (-46.4, 60.2) 0.807 | 22.6 (-19.1, 64.3) 0.324 | 2.0 (0.1, 44.4) 0.661 |
| Other | 3 (30.0%) | -3.3 (-34.2, 27.6) 0.839 | -2.0 (-43.8, 39.8) 0.928 | -0.0 (-48.8, 48.8) 1.000 | 11.7 (-9.5, 32.8) 0.315 | -21.3 (-69.4, 26.7) 0.413 | -18.7 (-75.7, 38.3) 0.541 | -1.7 (-46.2, 42.9) 0.944 | ––* |
| Version |  |  |  |  |  |  |  |  |  |
| First | 5 (50.0%) | Refrence | Refrence | Refrence | Refrence | Refrence | Refrence | Refrence | Refrence |
| Updated | 5 (50.0%) | 7.0 (-19.0, 33.0) 0.612 | 13.0 (-18.3, 44.3) 0.439 | 21.0 (-16.9, 58.9) 0.309 | 10.0 (-5.2, 25.2) 0.234 | 29.4 (-2.2, 61.0) 0.105 | 43.8 (12.1, 75.5) 0.027 | 26.8 (-4.1, 57.7) 0.127 | 2.7 (0.2, 45.1) 0.497 |
| Age range of target population |  |  |  |  |  |  |  |  |  |
| Adult | 3 (30.0%) | Refrence | Refrence | Refrence | Refrence | Refrence | Refrence | Refrence | Refrence |
| Children | 1 (10.0%) | 1.0 (-28.0, 30.0) 0.948 | 30.3 (-0.6, 61.3) 0.096 | 20.0 (-23.1, 63.1) 0.394 | 1.0 (-26.9, 28.9) 0.946 | -18.3 (-76.5, 39.9) 0.556 | 29.7 (-50.6, 109.9) 0.492 | 10.7 (-35.3, 56.7) 0.663 | ––* |
| All ages | 6 (60.0%) | -32.0 (-49.7, -14.3) 0.010 | -30.7 (-49.6, -11.7) 0.016 | -43.8 (-70.2, -17.4) 0.014 | -12.5 (-29.6, 4.6) 0.195 | -36.7 (-72.3, -1.0) 0.084 | -8.0 (-57.2, 41.2) 0.759 | -37.0 (-65.2, -8.8) 0.037 | ––* |
| Development method |  |  |  |  |  |  |  |  |  |
| CB | 4 (40.0%) | Refrence | Refrence | Refrence | Refrence | Refrence | Refrence | Refrence | Refrence |
| EB | 6 (60.0%) | 26.9 (7.4, 46.4) 0.027 | 36.7 (15.4, 58.1) 0.010 | 49.1 (25.5, 72.7) 0.004 | 23.1 (17.2, 29.0) <0.001 | 21.7 (-13.6, 57.0) 0.263 | 37.9 (1.6, 74.2) 0.075 | 45.8 (27.2, 64.3) 0.001 | ––* |
| Stated search dates |  |  |  |  |  |  |  |  |  |
| Yes | 7 (70.0%) | Refrence | Refrence | Refrence | Refrence | Refrence | Refrence | Refrence | Refrence |
| No | 3 (30.0%) | 2.5 (-26.3, 31.3) 0.868 | -11.9 (-46.4, 22.7) 0.520 | -16.2 (-59.0, 26.6) 0.478 | -10.4 (-27.1, 6.4) 0.259 | 4.0 (-36.9, 44.9) 0.851 | -33.1 (-75.2, 9.0) 0.162 | -17.4 (-54.8, 20.0) 0.388 | 1.2 (0.1, 22.9) 0.880 |
| Used CPG quality tool |  |  |  |  |  |  |  |  |  |
| Yes | 3 (30.0%) | Refrence | Refrence | Refrence | Refrence | Refrence | Refrence | Refrence | Refrence |
| No | 7 (70.0%) | -32.5 (-50.6, -14.5) 0.008 | -43.9 (-62.2, -25.5) 0.002 | -55.7 (-77.4, -34.0) 0.001 | -12.5 (-28.5, 3.6) 0.166 | -50.2 (-71.9, -28.6) 0.002 | -26.4 (-70.7, 17.8) 0.276 | -45.9 (-69.0, -22.8) 0.005 | ––* |
| Included CPG methodologist |  |  |  |  |  |  |  |  |  |
| Yes | 1 (10.0%) | Refrence | Refrence | Refrence | Refrence | Refrence | Refrence | Refrence | Refrence |
| No | 4 (40.0%) | -18.2 (-55.8, 19.3) 0.372 | -17.3 (-67.0, 32.5) 0.519 | -17.5 (-81.6, 46.6) 0.609 | -16.5 (-45.2, 12.2) 0.298 | -32.2 (-77.8, 13.3) 0.208 | 12.0 (-67.7, 91.7) 0.777 | -18.3 (-76.8, 40.3) 0.561 | ––* |
| Not stated | 5 (50.0%) | -37.6 (-74.4, -0.8) 0.085 | -39.6 (-88.3, 9.1) 0.155 | -44.6 (-107.4, 18.2) 0.206 | -9.6 (-37.8, 18.6) 0.525 | -63.2 (-107.8, -18.6) 0.027 | -6.6 (-84.7, 71.5) 0.873 | -39.0 (-96.4, 18.4) 0.224 | ––* |
| Used grading system |  |  |  |  |  |  |  |  |  |
| Yes | 6 (60.0%) | Refrence | Refrence | Refrence | Refrence | Refrence | Refrence | Refrence | Refrence |
| No | 4 (40.0%) | -26.9 (-46.4, -7.4) 0.027 | -36.7 (-58.1, -15.4) 0.010 | -49.1 (-72.7, -25.5) 0.004 | -23.1 (-29.0, -17.2) <0.001 | -21.7 (-57.0, 13.6) 0.263 | -37.9 (-74.2, -1.6) 0.075 | -45.8 (-64.3, -27.2) 0.001 | ––* |
| With funding sources |  |  |  |  |  |  |  |  |  |
| Yes | 4 (40.0%) | Refrence | Refrence | Refrence | Refrence | Refrence | Refrence | Refrence | Refrence |
| No | 4 (40.0%) | -9.0 (-38.9, 20.9) 0.574 | -5.7 (-41.6, 30.1) 0.763 | 0.7 (-44.3, 45.8) 0.975 | 4.0 (-15.2, 23.2) 0.695 | 1.0 (-37.9, 39.9) 0.961 | 45.5 (8.8, 82.2) 0.045 | 9.7 (-29.1, 48.6) 0.638 | 0.3 (0.0, 6.7) 0.472 |
| Not stated | 2 (20.0%) | -16.2 (-52.9, 20.4) 0.413 | -24.5 (-68.5, 19.5) 0.311 | -26.8 (-81.9, 28.4) 0.373 | -5.0 (-28.5, 18.5) 0.689 | -33.2 (-80.9, 14.4) 0.214 | -2.3 (-47.1, 42.6) 0.924 | -21.0 (-68.5, 26.5) 0.415 | ––* |

*The model failed because of the small sample size; AGREE: Appraisal of Guidelines for Research and Evaluation; CPG: clinical practice guideline; CI: confidence interval; OR: odds ratio; MND: motor neuron disease; EB: evidence-based; CB: consensus-based.

**Reference**

1. Pitarch CI, Cabrera-Serrano M, Calvo MR, Cattinari MG, Espinosa GS, Fernandez-Ramos JA, et al. Delphi consensus on recommendations for the treatment of spinal muscular atrophy in Spain (RET-AME consensus). *Neurologia (Engl Ed)* (2022) 37(3):216-28. 10.1016/j.nrleng.2021.07.002.

2. Georges M, Perez T, Rabec C, Jacquin L, Finet-Monnier A, Ramos C, et al. Proposals from a French expert panel for respiratory care in ALS patients. *Respir Med Res-Franc* (2022) 81:100901. 10.1016/j.resmer.2022.100901.

3. Shoesmith C, Abrahao A, Benstead T, Chum M, Dupre N, Izenberg A, et al. Canadian best practice recommendations for the management of amyotrophic lateral sclerosis. *Can Med Assoc J* (2020) 192(46): E1453-68. 10.1503/cmaj.191721.

4. Corcia P, Lumbroso S, Cazeneuve C, Mouzat K, Camu W, Vourc'H P. Pre-symptomatic diagnosis in ALS. *Rev Neurol-France* (2020) 176(3):166-9. 10.1016/j.neurol.2019.07.027.

5. Rimmer KP, Kaminska M, Nonoyama M, Giannouli E, Maltais F, Morrison DL, et al. Home mechanical ventilation for patients with Amyotrophic Lateral Sclerosis: A Canadian Thoracic Society clinical practice guideline. *Canadian Journal of Respiratory, Critical Care, and Sleep Medicine* (2019) 3(1):9-27. 10.1080/24745332.2018.1559644.

6. Pattee GL, Plowman EK, Focht GK, Costello J, Brooks BR, Berry JD, et al. Provisional best practices guidelines for the evaluation of bulbar dysfunction in amyotrophic lateral sclerosis. *Muscle Nerve* (2019) 59(5):531-6. 10.1002/mus.26408.

7. Silvinato A, Bernardo WM. Spinal muscular atrophy 5Q - Treatment with nusinersen. *Rev Assoc Med Bras* (2018) 64(6):484-91. 10.1590/1806-9282.64.06.484.

8. Finkel RS, Mercuri E, Meyer OH, Simonds AK, Schroth MK, Graham RJ, et al. Diagnosis and management of spinal muscular atrophy: Part 2: Pulmonary and acute care; medications, supplements and immunizations; other organ systems; and ethics. *Neuromuscular Disord* (2018) 28(3):197-207. 10.1016/j.nmd.2017.11.004.

9. Mercuri E, Finkel RS, Muntoni F, Wirth B, Montes J, Main M, et al. Diagnosis and management of spinal muscular atrophy: Part 1: Recommendations for diagnosis, rehabilitation, orthopedic and nutritional care. *Neuromuscular Disord* (2018) 28(2):103-15. 10.1016/j.nmd.2017.11.005.

10. *Motor neurone disease: assessment and management*.London: National Institute for Health and Care Excellence (NICE) (2019).

11. Cuisset JM, Estournet B. Recommendations for the diagnosis and management of typical childhood spinal muscular atrophy. *Rev Neurol-France* (2012) 168(12):902-9. 10.1016/j.neurol.2012.07.020.

12. Andersen PM, Abrahams S, Borasio GD, de Carvalho M, Chio A, Van Damme P, et al. EFNS guidelines on the clinical management of amyotrophic lateral sclerosis (MALS)--revised report of an EFNS task force. *Eur J Neurol* (2012) 19(3):360-75. 10.1111/j.1468-1331.2011.03501. x.

13. Filippi M, Agosta F, Abrahams S, Fazekas F, Grosskreutz J, Kalra S, et al. EFNS guidelines on the use of neuroimaging in the management of motor neuron diseases. *Eur J Neurol* (2010) 17(4):520-6. 10.1111/j.1468-1331.2010.02951. x.

14. Miller RG, Jackson CE, Kasarskis EJ, England JD, Forshew D, Johnston W, et al. Practice parameter update: the care of the patient with amyotrophic lateral sclerosis: drug, nutritional, and respiratory therapies (an evidence-based review): report of the Quality Standards Subcommittee of the American Academy of Neurology. *Neurology* (2009) 73(15):1218-26. 10.1212/WNL.0b013e3181bc0141.

15. Lo JK, Robinson LR. Post-polio syndrome and the late effects of poliomyelitis: Part 2. treatment, management, and prognosis. *Muscle Nerve* (2018) 58(6):760-9. 10.1002/mus.26167.

16. ACOG committee opinion No. 432: spinal muscular atrophy. *Obstet Gynecol* (2009) 113(5):1194-6. 10.1097/AOG.0b013e3181a6d03a. PubMed PMID: 19384151.

17. de Carvalho M, Dengler R, Eisen A, England JD, Kaji R, Kimura J, et al. Electrodiagnostic criteria for diagnosis of ALS. *Clin Neurophysiol* (2008) 119(3):497-503. 10.1016/j.clinph.2007.09.143.
